# Supplementary material for: Habitat openness and squamate color evolution over deep time
Source: Nat Commun. 2025 Mar 17;16:2625. doi: 10.1038/s41467-025-57547-6 (PMC11914068; doi:10.1038/s41467-025-57547-6)
Supplement: Supplementary file 1 — Supplementary Information [file 41467_2025_57547_MOESM1_ESM.pdf]

Supplementary Materials for

Habitat openness and squamate color evolution over deep time

Jonathan Goldenberg, Karen Bisschop, Joshua W. Lambert, Michaël Nicolaï, Rampal S. Etienne, Liliana D'Alba, Matthew D. Shawkey

Corresponding author: jonathan.goldenberg@ugent.be

The PDF file includes:

- Supplementary Figures 1-15 support the associations and relationship between brightness and analyzed ecological and environmental data (p. 2-16).
- Supplementary Figure 16 compares mean brightness evolutionary rates of focal clades with changes in foraminiferal $\delta^{18}O$ (p. 17).
- Supplementary Figure 17 shows the correlation analyses between brightness evolution rates and $\delta^{18}O$ (p. 18).
- Supplementary Figure 18 compares brightness evolution rates across habitat types (p. 19).
- Supplementary Figures 19-25 show phylogenetic reconstructions mapped with brightness evolutionary rates along branches at family level (p. 20-26).
- Supplementary Figure 26 shows variations in brightness variance within and among species (p. 27).
- Supplementary Figures 27-33 evaluate the robustness of the evolutionary rate analyses (p. 28-34).
- Supplementary Figure 34 supports the choice of altitude clusters (p. 35).
- Supplementary Figure 35 fits a model to study how evolutionary rates change over time in response to $\delta^{18}O$ (p. 36).
- Supplementary Table 1 compares evolutionary rates between focal clades (p. 37).
- Supplementary Table 2 reports model evaluation power between different subsets (p. 38).
- Supplementary References (p. 39).

Supplementary Figure 1

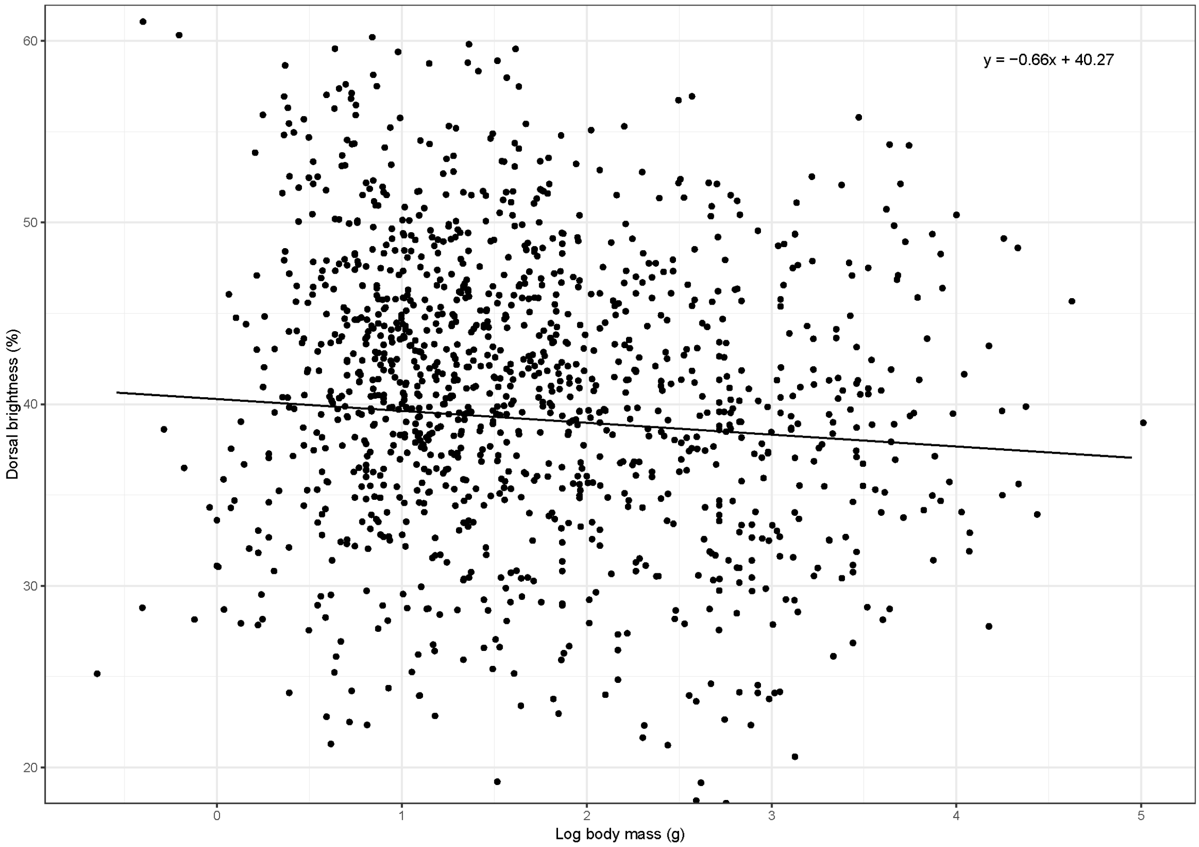

Relationship between body mass and dorsal brightness. Predicted relationship between body mass values and dorsal brightness from the MCMCglmm analysis at total level (sample size *n*: 1249). Dots indicate species' raw body size values.

Supplementary Figure 2

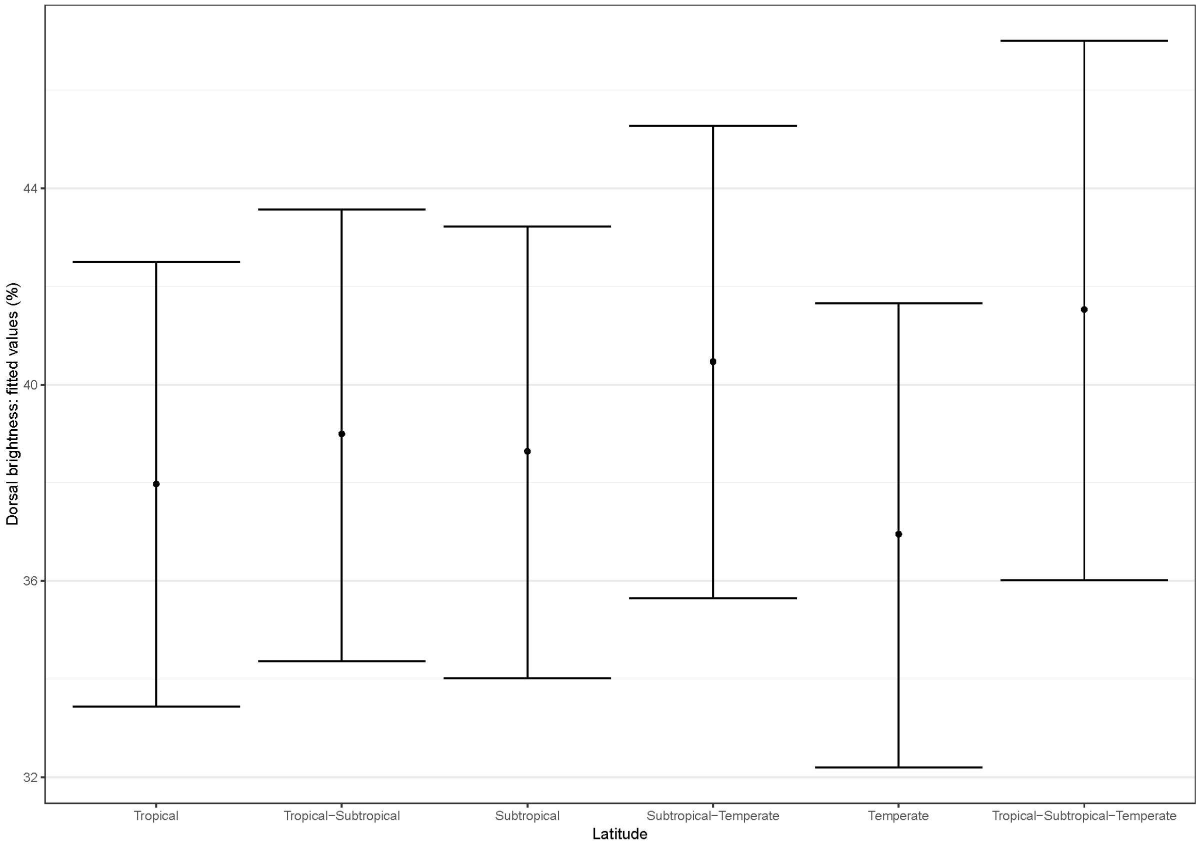

Correlation between latitude and dorsal brightness. Predicted correlation between latitudinal distribution and dorsal brightness from the MCMCglmm analysis at total level (sample size *n*: 1249). Bars represent 95% credible intervals. Dots indicate mean predicted values.

Supplementary Figure 3

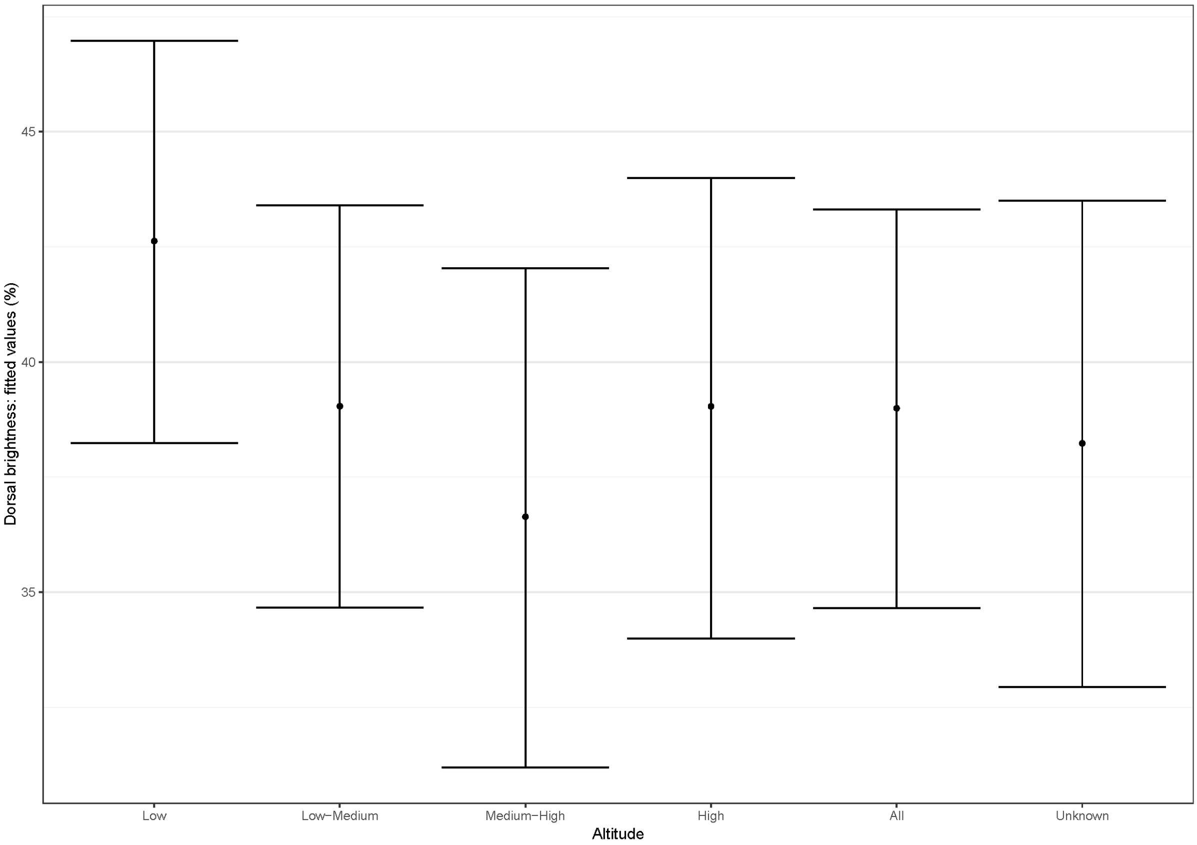

Correlation between altitude and dorsal brightness. Predicted correlation between altitudinal variations and dorsal brightness from the MCMCglmm analysis at total level (sample size *n*: 1249). Bars represent 95% credible intervals. Dots indicate mean predicted values.

Supplementary Figure 4

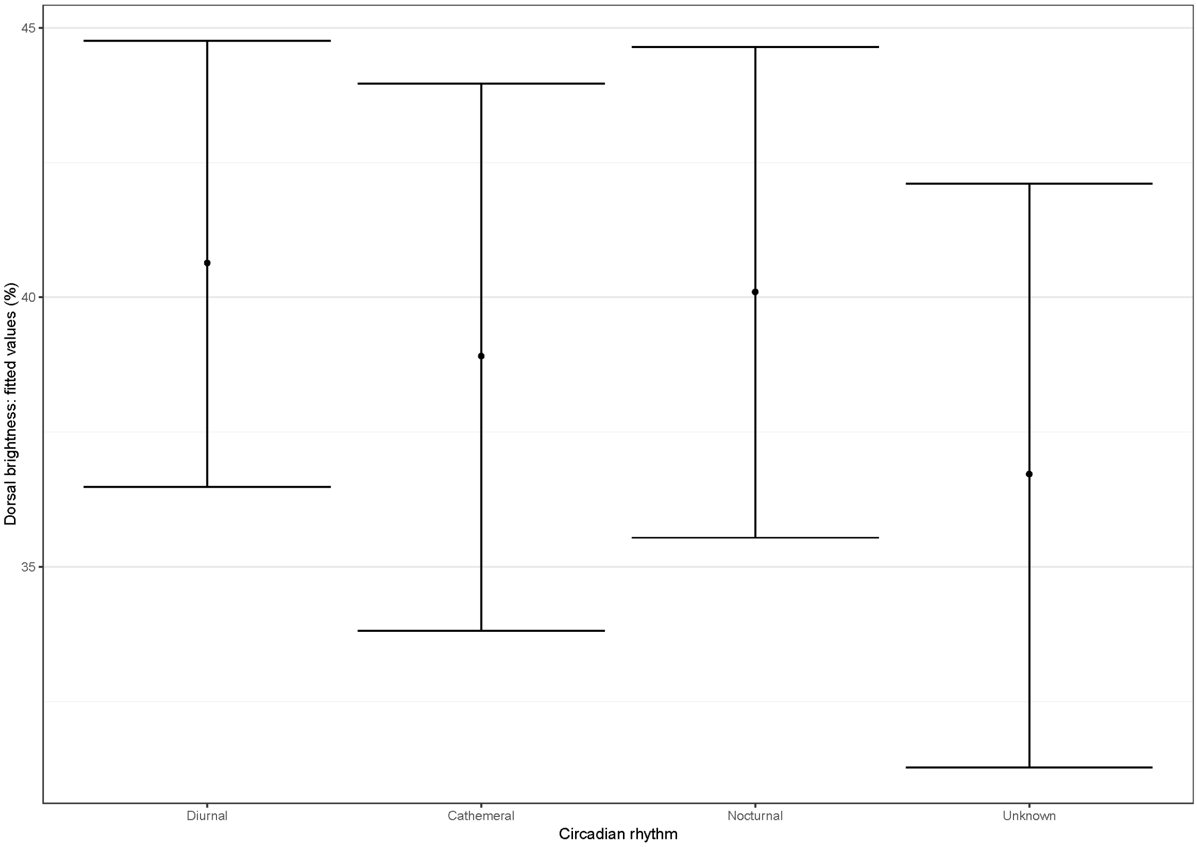

Correlation between circadian rhythm and dorsal brightness. Predicted correlation between circadian rhythm and dorsal brightness from the MCMCglmm analysis at total level (sample size *n*: 1249). Bars represent 95% credible intervals. Dots indicate mean predicted values.

Supplementary Figure 5

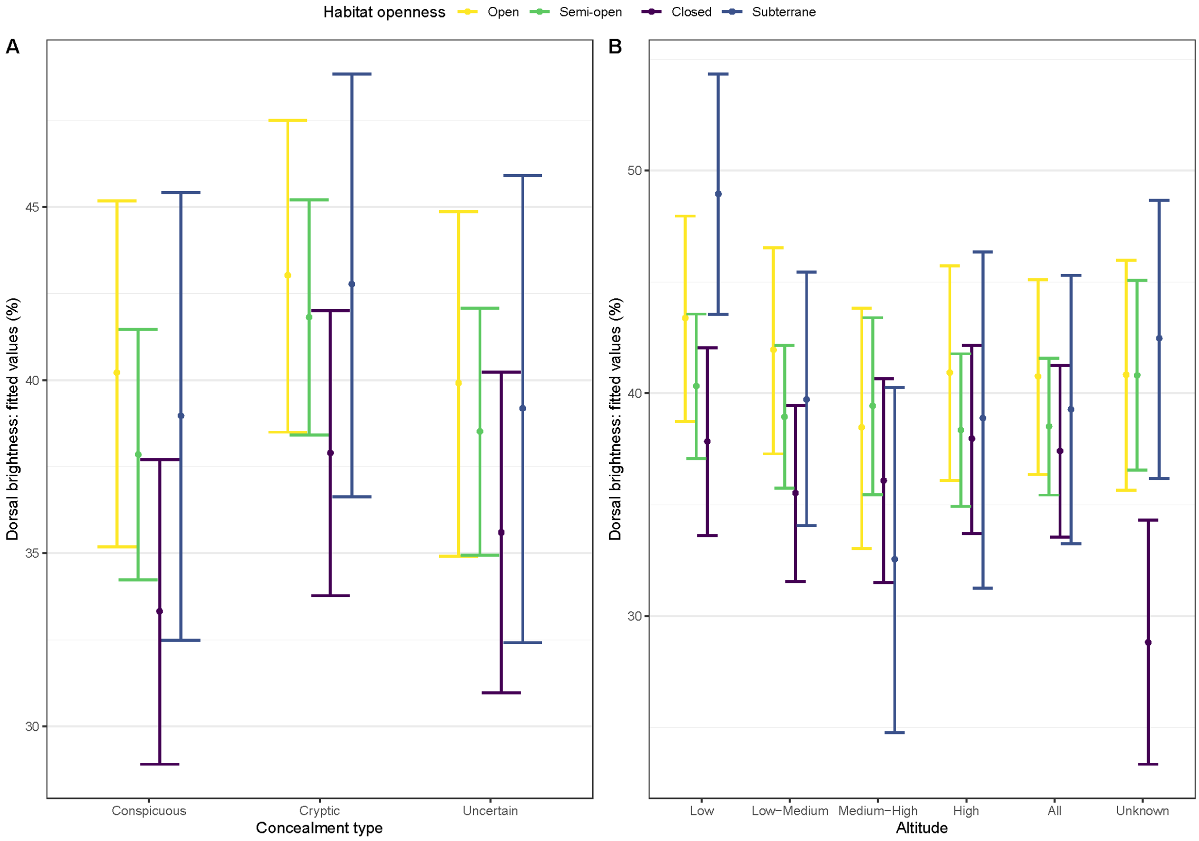

Three-way interaction plots between concealment type, altitude, habitat openness, and dorsal brightness. Predicted correlation effects between (**A**) between concealment type and habitat openness, and (**B**) altitudinal variation and habitat openness on shaping dorsal brightness from the MCMCglmm analysis at total level (sample size *n*: 1249).

Supplementary Figure 6

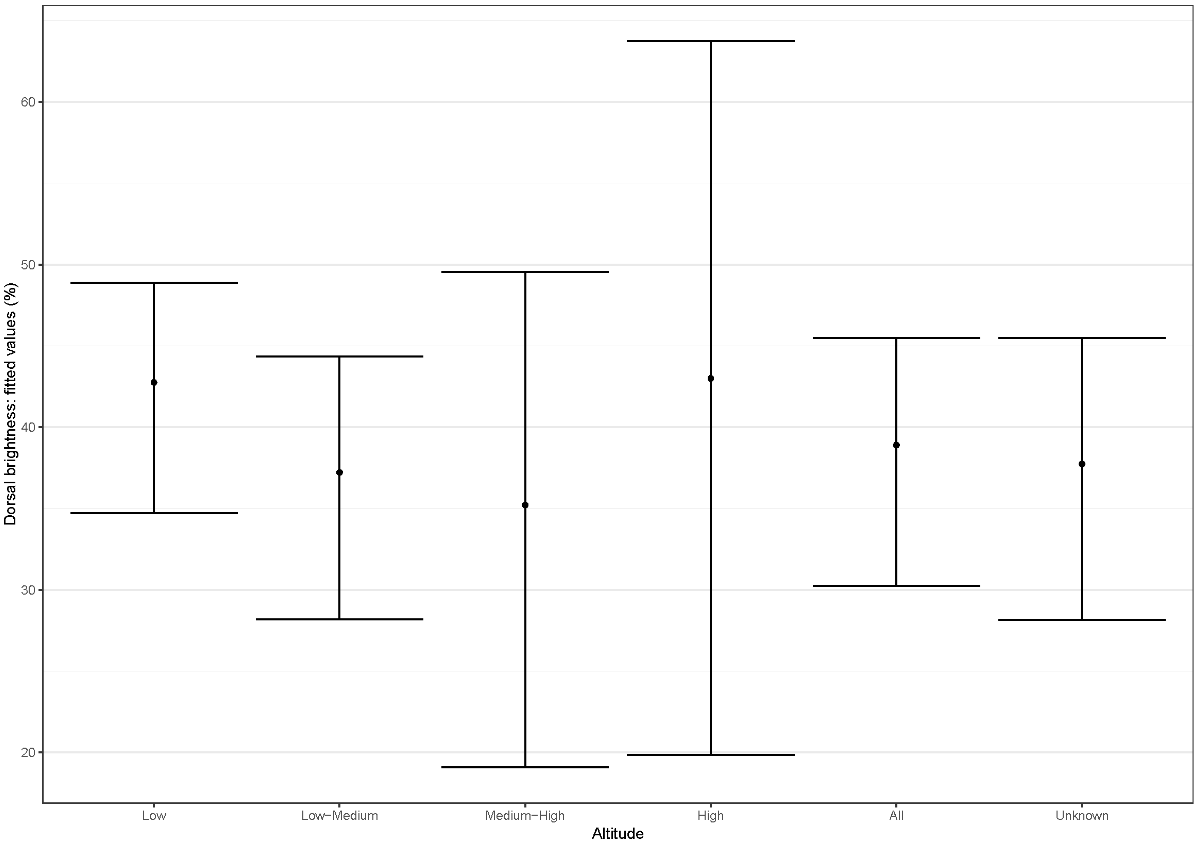

Correlation between altitude and dorsal brightness in Scincidae. Predicted correlation between elevation range and dorsal brightness from the MCMCglmm analysis at Scincidae level (sample size *n*: 115). Bars represent 95% credible intervals. Dots indicate mean predicted values.

Supplementary Figure 7

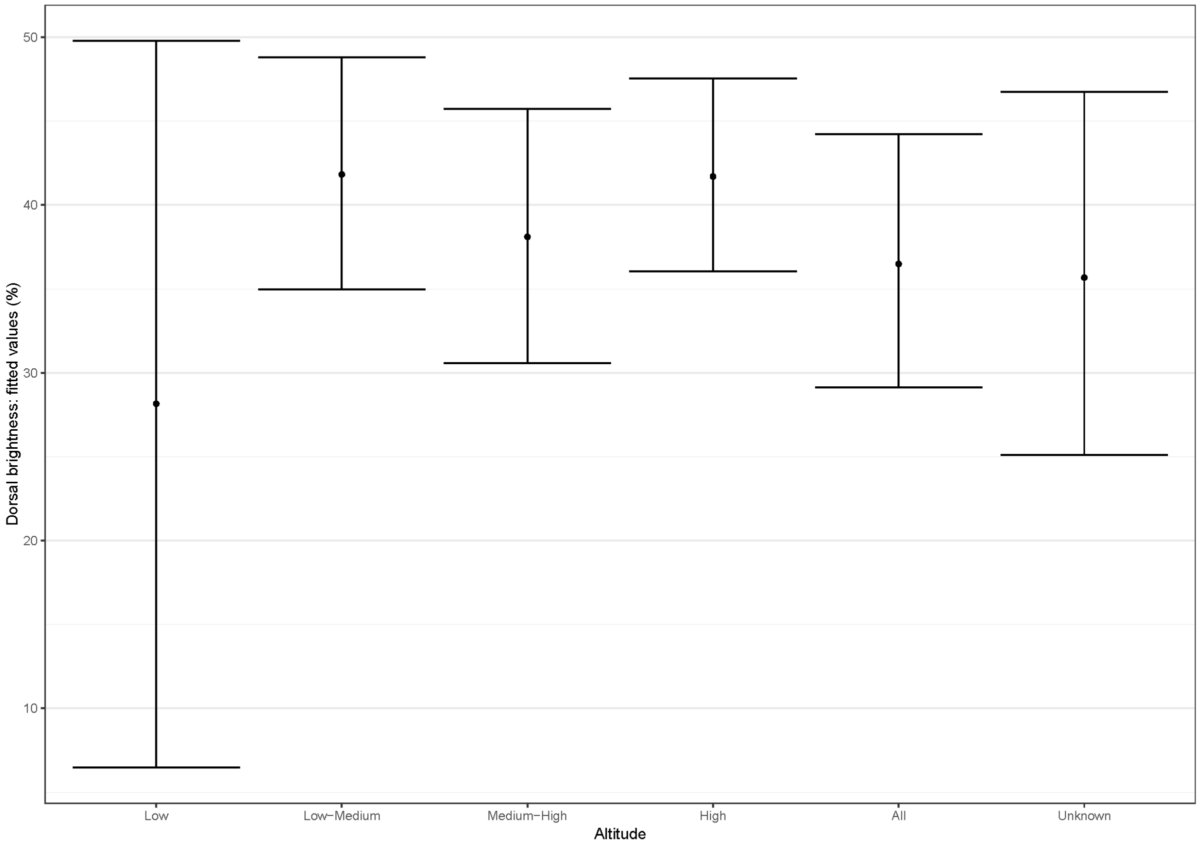

Correlation between altitude and dorsal brightness in Cordylidae. Predicted correlation between elevation range and dorsal brightness from the MCMCglmm analysis at Cordylidae level (sample size *n*: 42). Bars represent 95% credible intervals. Dots indicate mean predicted values.

Supplementary Figure 8

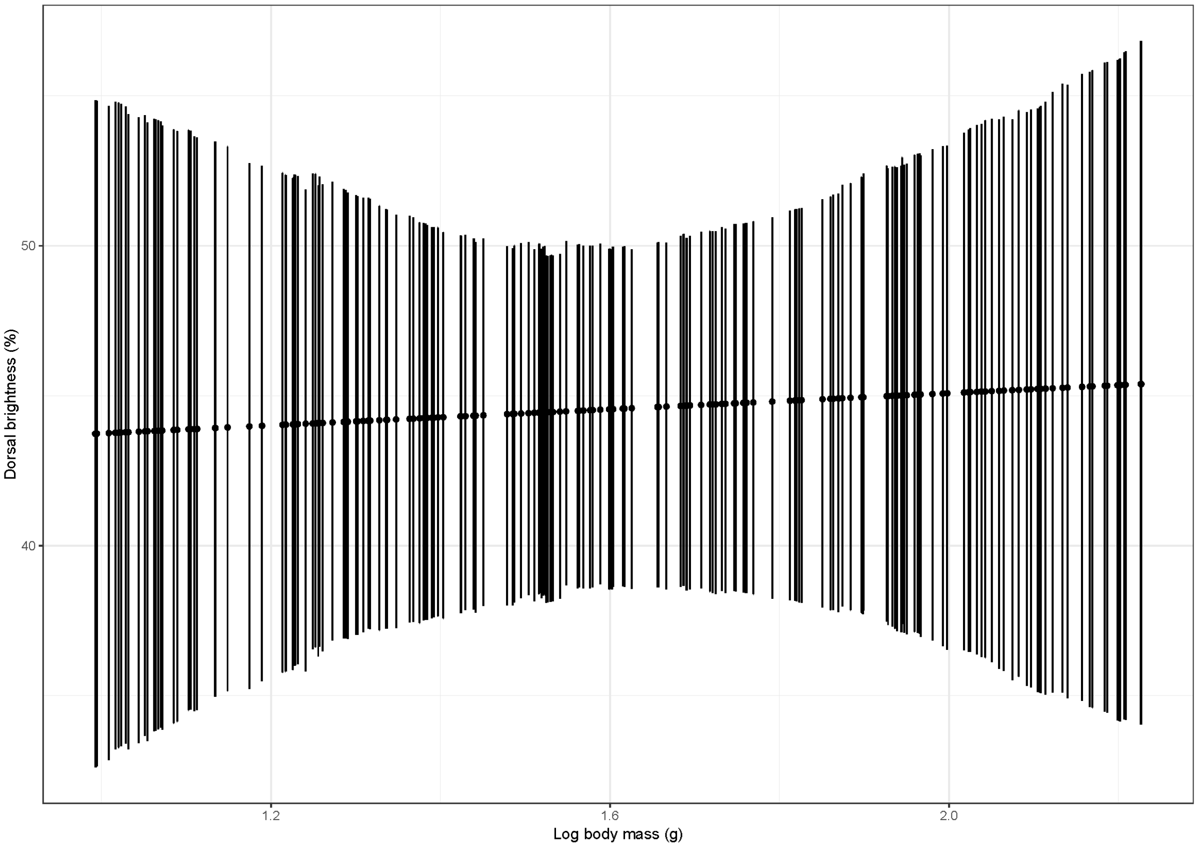

Relationship between body mass and dorsal brightness in Tropiduridae. Predicted relationship between body mass data and dorsal brightness from the MCMCglmm analysis at Tropiduridae level (sample size *n*: 70). Bars represent 95% credible intervals. Dots indicate mean predicted values.

Supplementary Figure 9

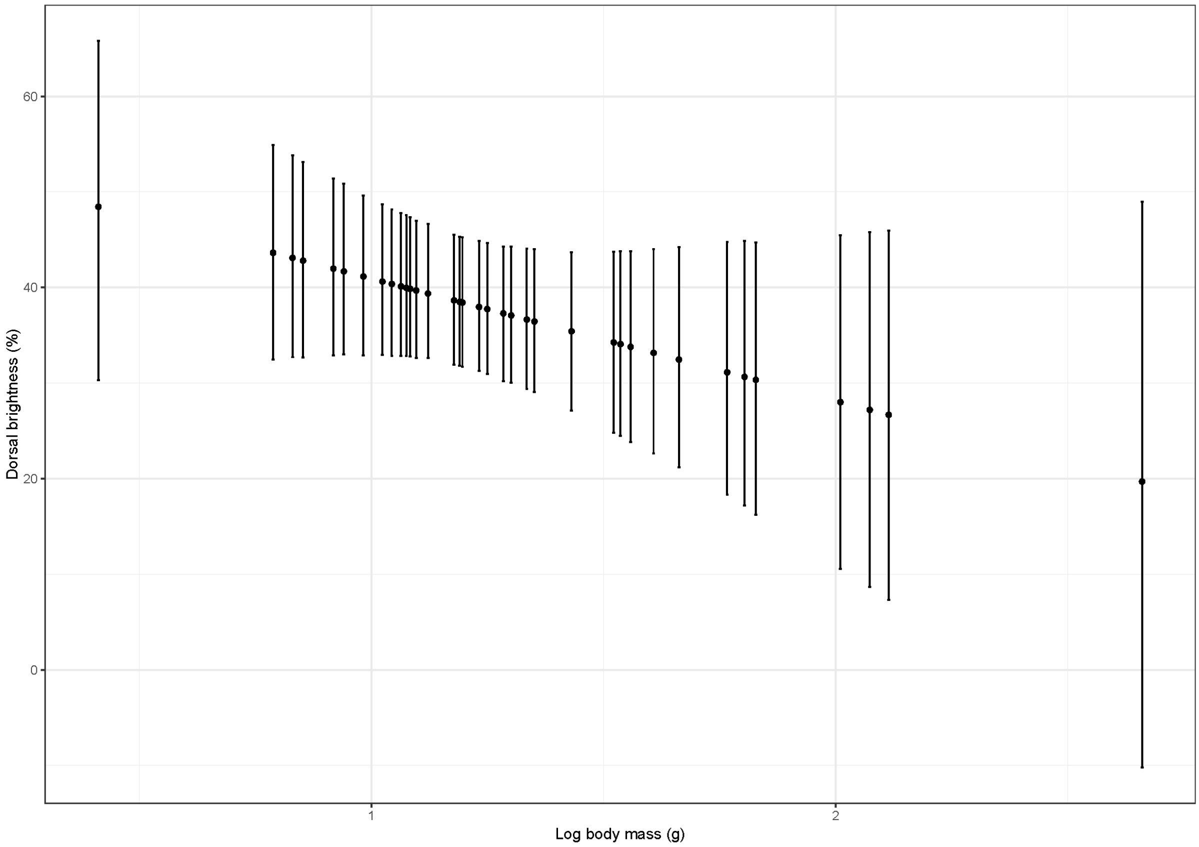

Relationship between body mass and dorsal brightness in Cordylidae. Predicted relationship between body mass data and dorsal brightness from the MCMCglmm analysis at Cordylidae level (sample size *n*: 42). Bars represent 95% credible intervals. Dots indicate mean predicted values.

Supplementary Figure 10

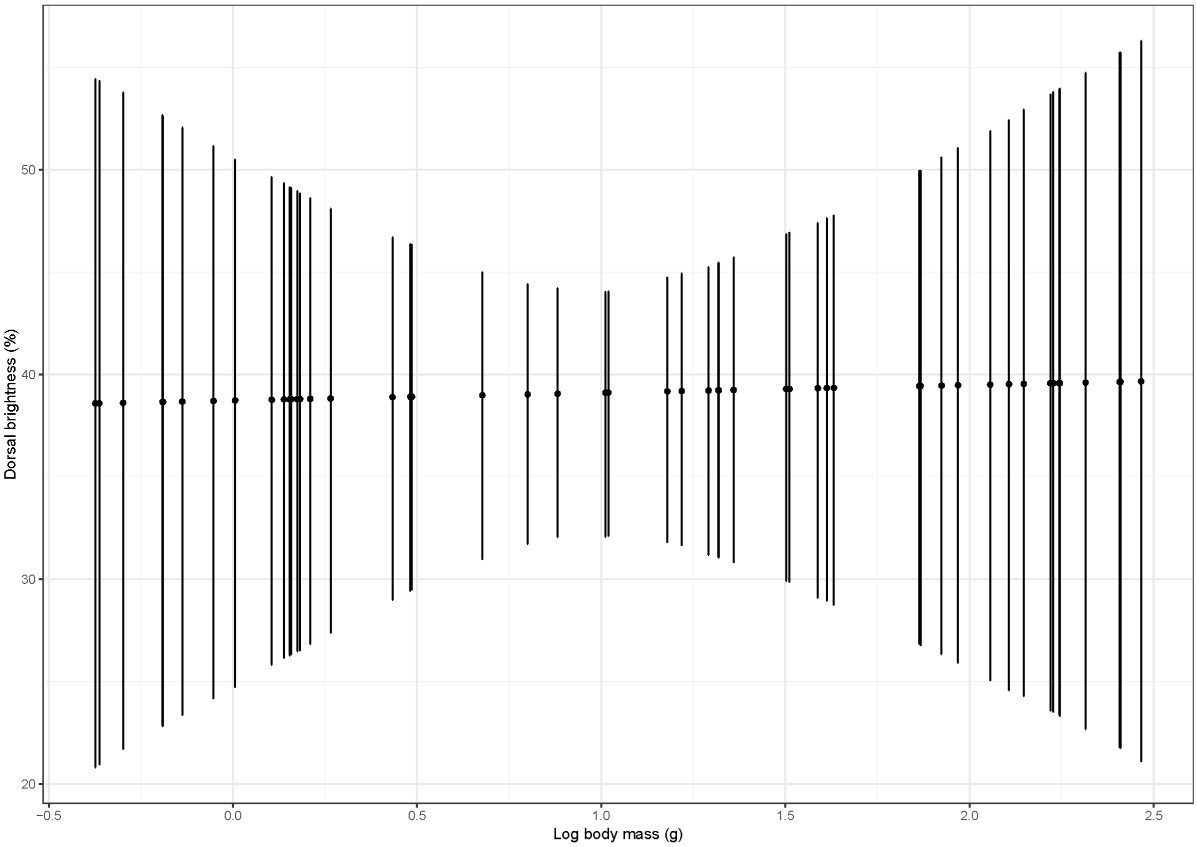

Relationship between body mass and dorsal brightness in Scincidae. Predicted relationship between body mass data and dorsal brightness from the MCMCglmm analysis at Scincidae level (sample size *n*: 115). Bars represent 95% credible intervals. Dots indicate mean predicted values.

Supplementary Figure 11

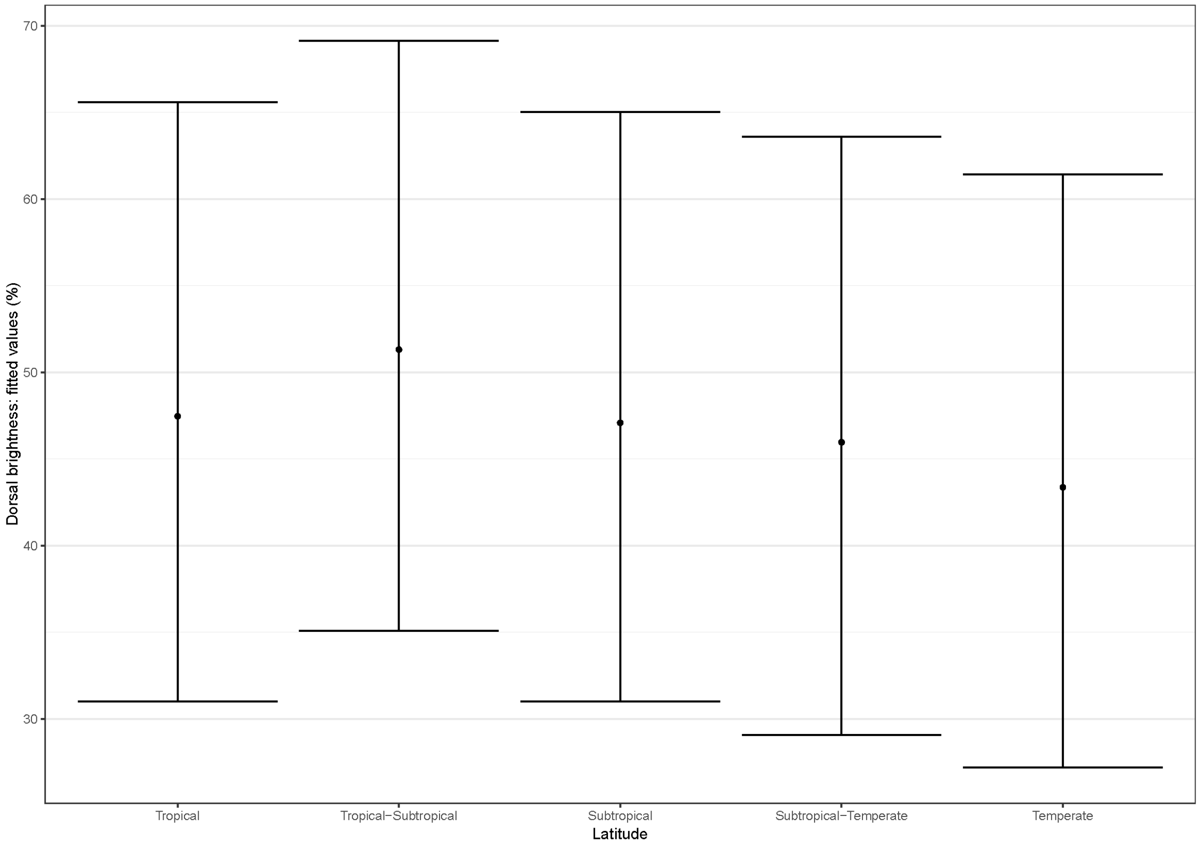

Correlation between latitude and dorsal brightness in Lacertidae. Predicted correlation between latitudinal distribution and dorsal brightness from the MCMCglmm analysis at Lacertidae level (sample size *n*: 184). Bars represent 95% credible intervals. Dots indicate mean predicted values.

Supplementary Figure 12

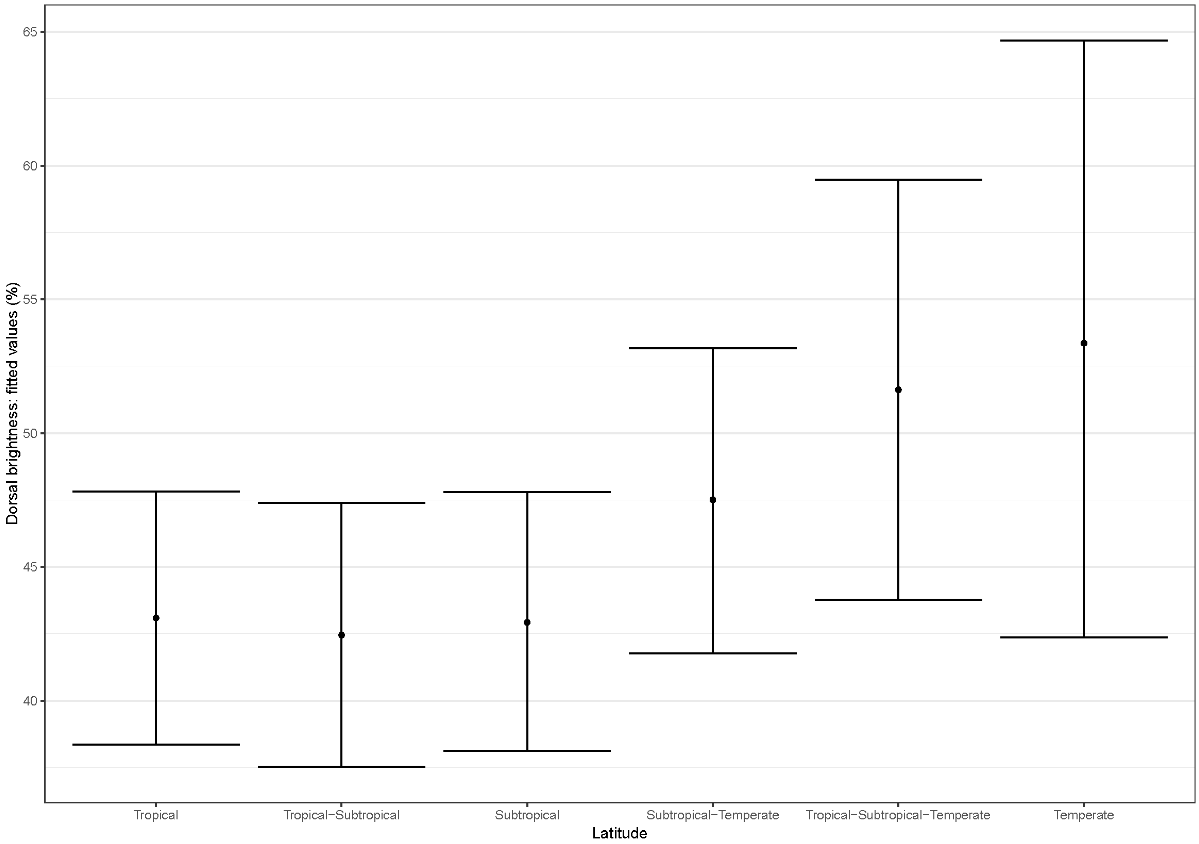

Correlation between latitude and dorsal brightness in Phrynosomatidae. Predicted correlation between latitudinal distribution and dorsal brightness from the MCMCglmm analysis at Phrynosomatidae level (sample size *n*: 111). Bars represent 95% credible intervals. Dots indicate mean predicted values.

Supplementary Figure 13

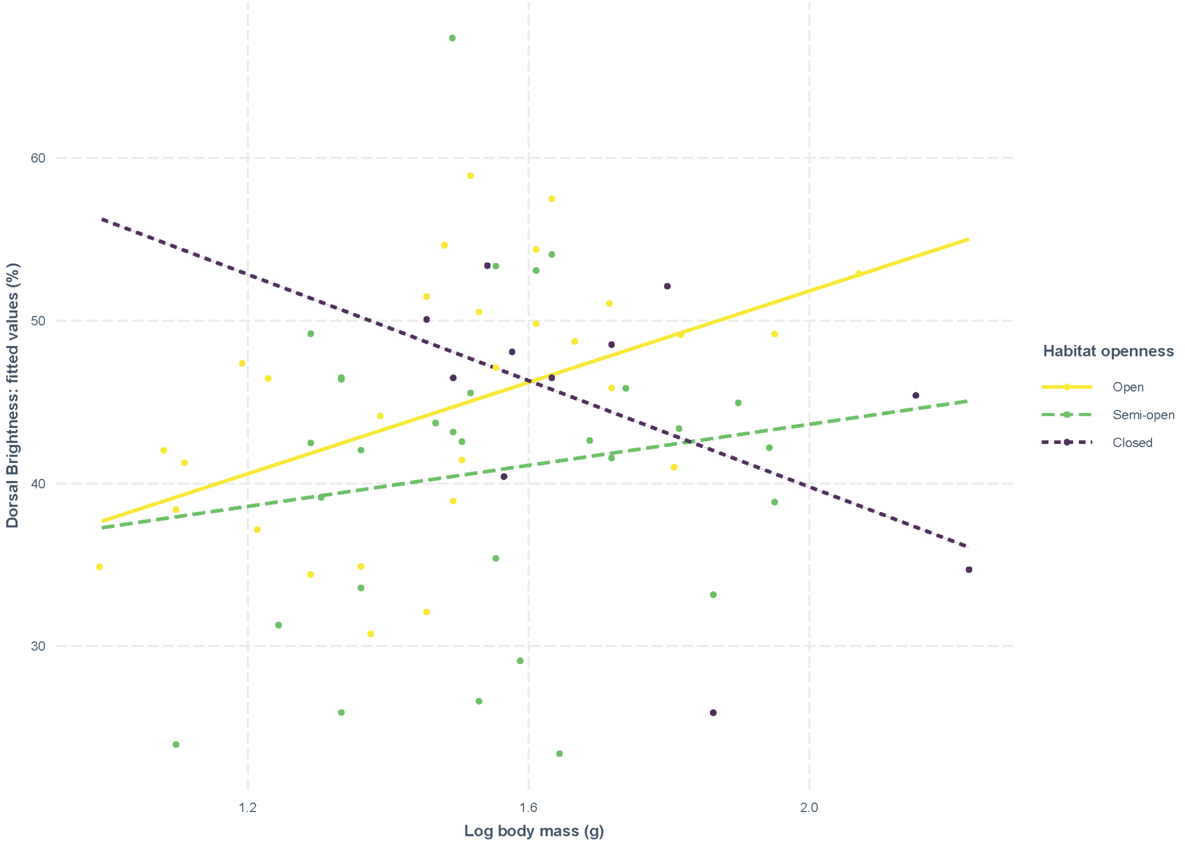

Correlation between body mass, habitat openness, and dorsal brightness in Tropiduridae. Three-way interaction plot between the fitted dorsal brightness values from the MCMCglmm analysis at Tropiduridae level (sample size *n*: 70), log body mass, and habitat openness. Data points depict individual species clustered (i.e. colored) by their habitat openness class.

Supplementary Figure 14

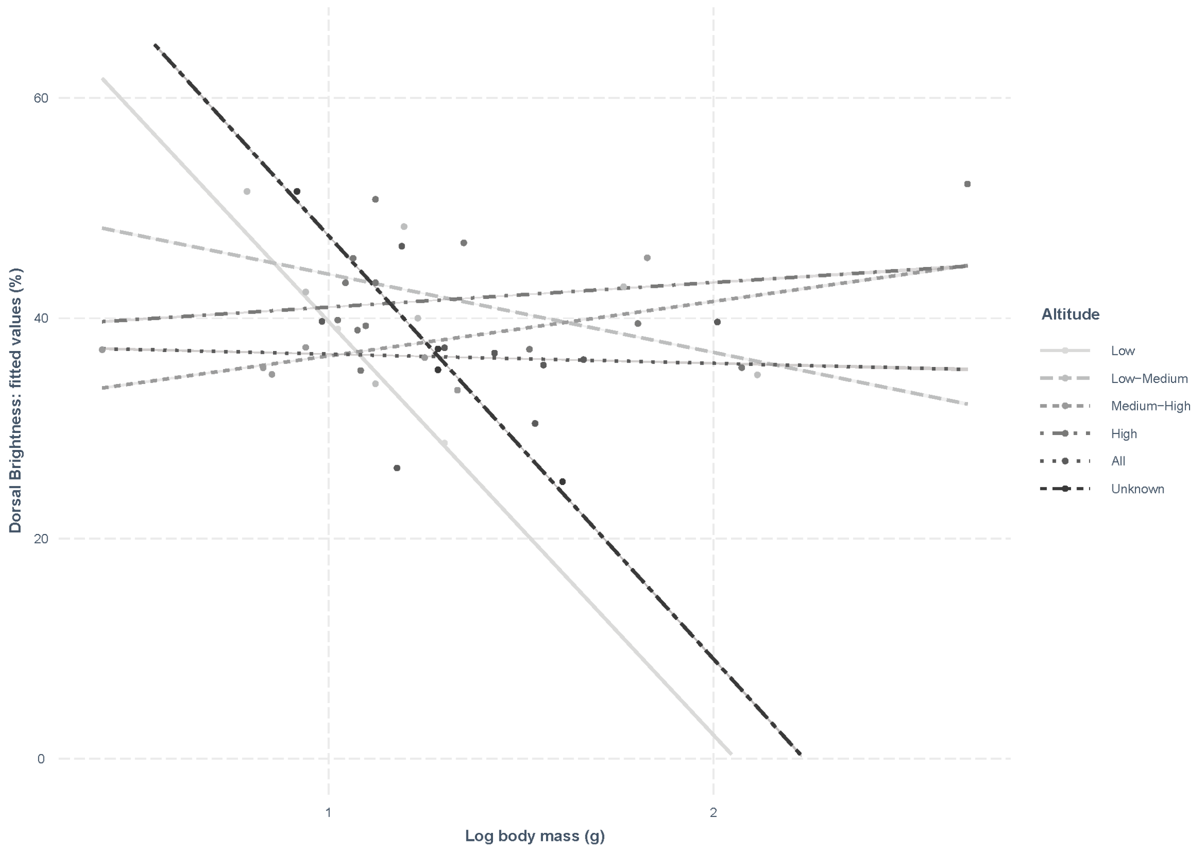

Correlation between body mass, altitude, and dorsal brightness in Cordylidae. Three-way interaction plot between the fitted dorsal brightness values from the MCMCglmm analysis at Cordylidae level (sample size *n*: 42), log body mass, and altitude. Data points depict individual species clustered (i.e. colored) by their altitudinal distribution.

Supplementary Figure 15

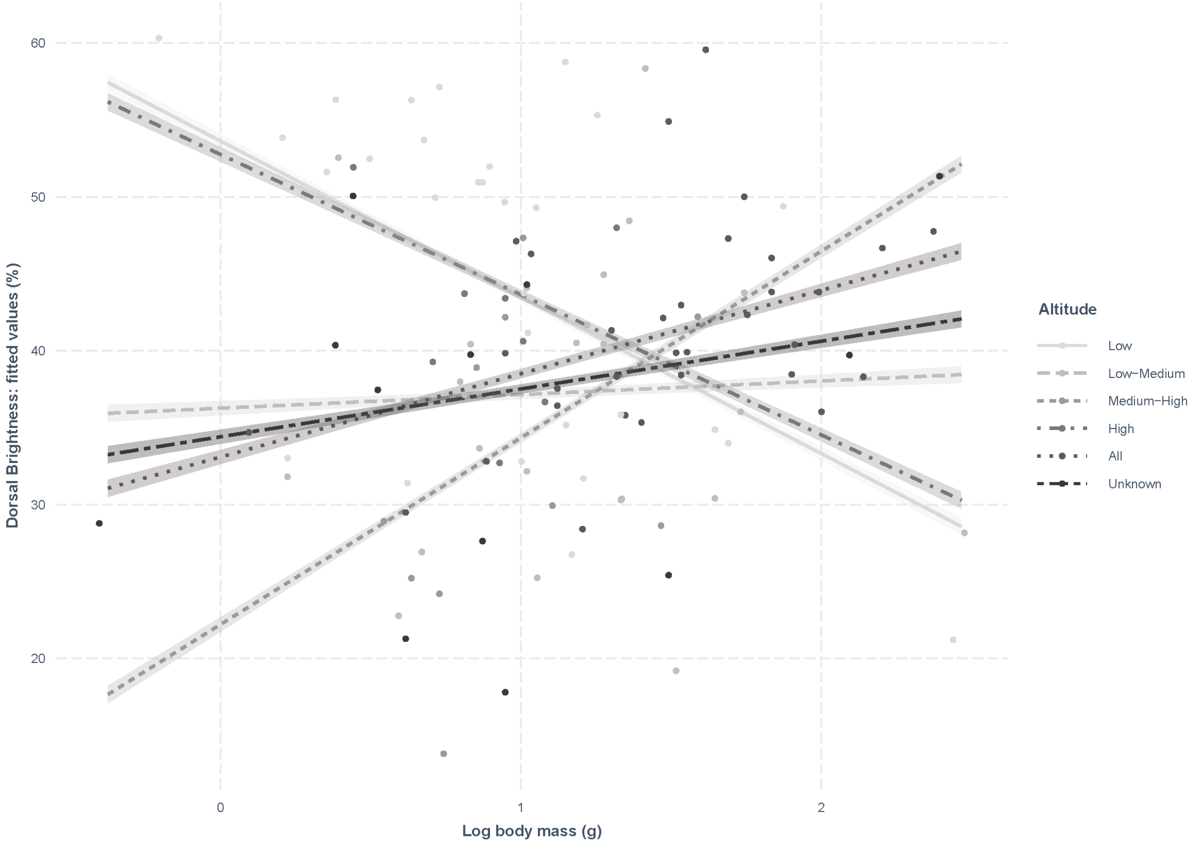

Correlation between body mass, altitude, and dorsal brightness in Scincidae. Three-way interaction plot between the fitted dorsal brightness values from the MCMCglmm analysis at Scincidae level (sample size *n*: 115), log body mass, and altitude. Data points depict individual species clustered (i.e. colored) by their altitudinal distribution.

Supplementary Figure 16

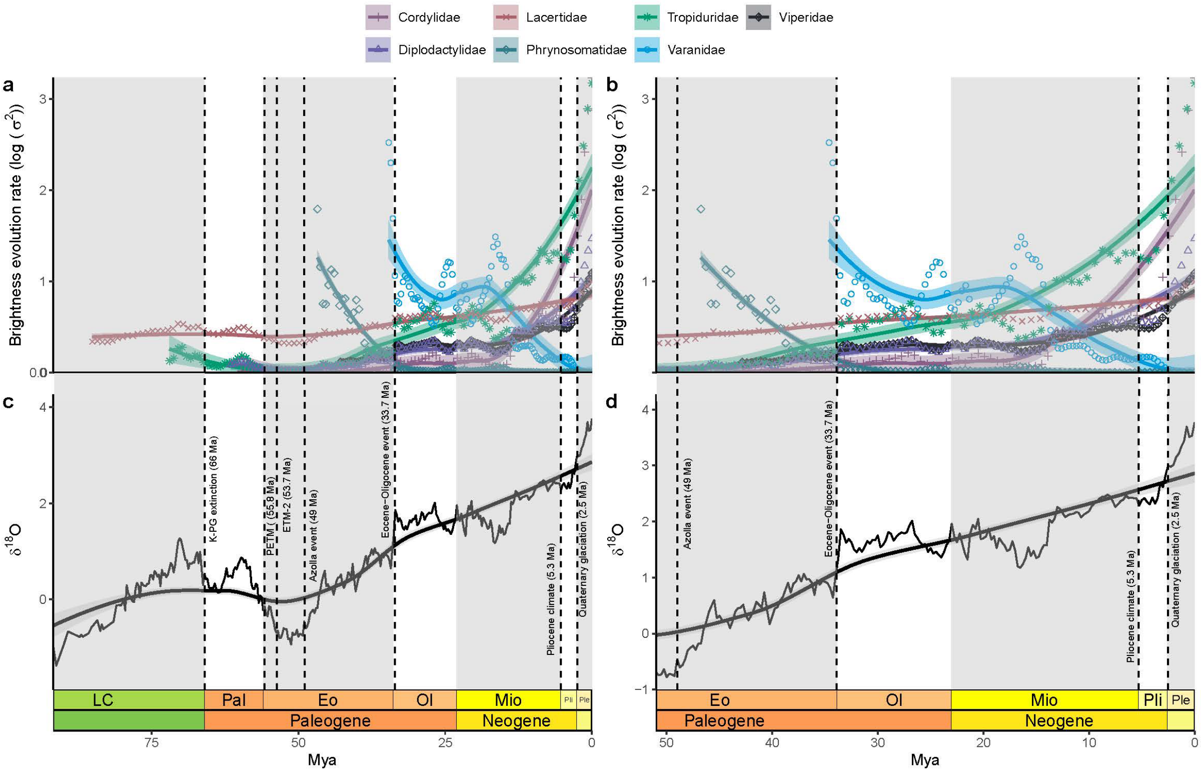

Brightness evolution rates in squamates. On the left panels (**a**, **c**) color brightness evolution rates (σ^2) accounting for measurement error for each clade, since their roots, are plotted against $\delta^{18}O$ (our proxy for habitat openness change; data from Gaskell et al.[1]). On the right panels (**b**, **d**) data are zoomed into the Eocene - Pliocene interface. Dashed lines indicate major global climate shift events induced by geological or environmental processes. Epochs, periods, and their acronyms and color codes follow the International Geological Time Scale as set by the International Commission on Stratigraphy (ICS). Individual data points are grouped by shape at family level and show brightness evolution rates at nodes. Shaded areas around solid curves show 95% C.I. Sample size (*n*): Cordylidae = 42; Diplodactylidae = 91; Lacertidae = 184; Phrynosomatidae = 111; Tropiduridae = 70; Varanidae = 53; Viperidae = 206.

Supplementary Figure 17

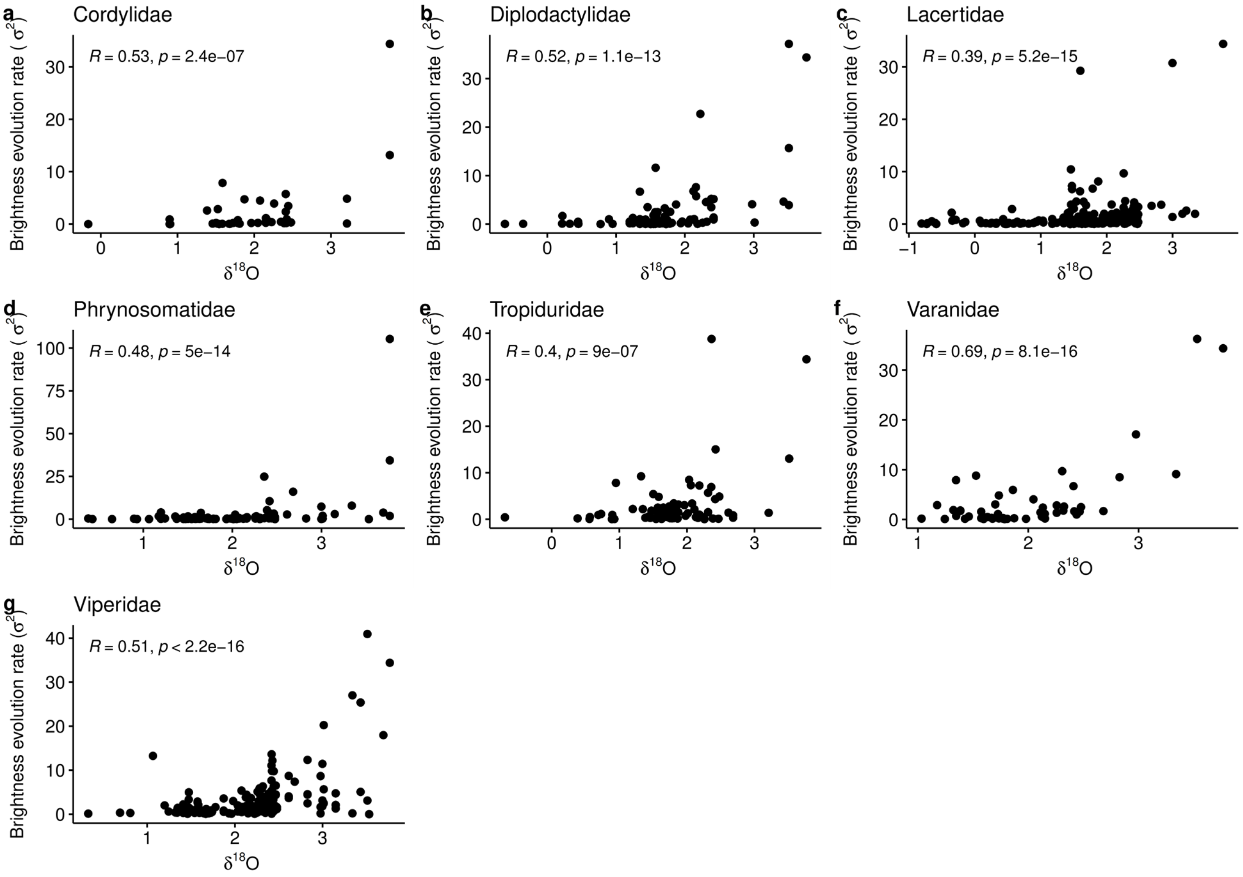

Correlation analyses between brightness evolution rates (σ^2) and δ^{18}O. Each panel reports the correlation between σ^2 and δ^{18}O across the focal families. When assumptions of data distribution were not met (i.e. Lacertidae, Phrynosomatidae, Viperidae) a Spearman correlation was applied instead of Pearson's. Points indicate data (i.e. σ^2 and δ^{18}O) at nodes of the phylogeny. Sample size (*n*): Cordylidae = 42; Diplodactylidae = 91; Lacertidae = 184; Phrynosomatidae = 111; Tropiduridae = 70; Varanidae = 53; Viperidae = 206.

Supplementary Figure 18

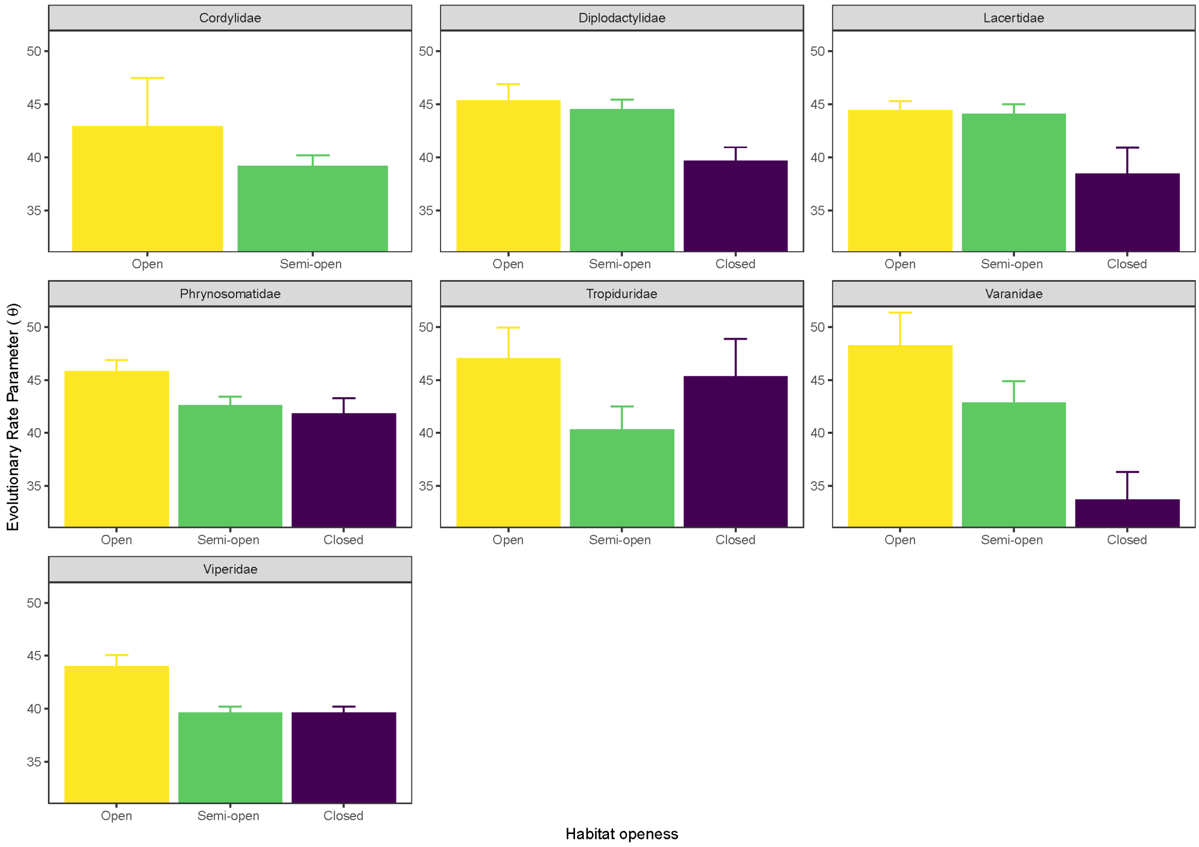

Evolutionary rate parameter. Each panel reports θ values of brightness evolution (+ standard error) in different habitat types across the focal families under an Ornstein-Uhlenbeck model of evolution. Colors designate different habitat types as in Fig.2 in the main text.

Supplementary Figure 19

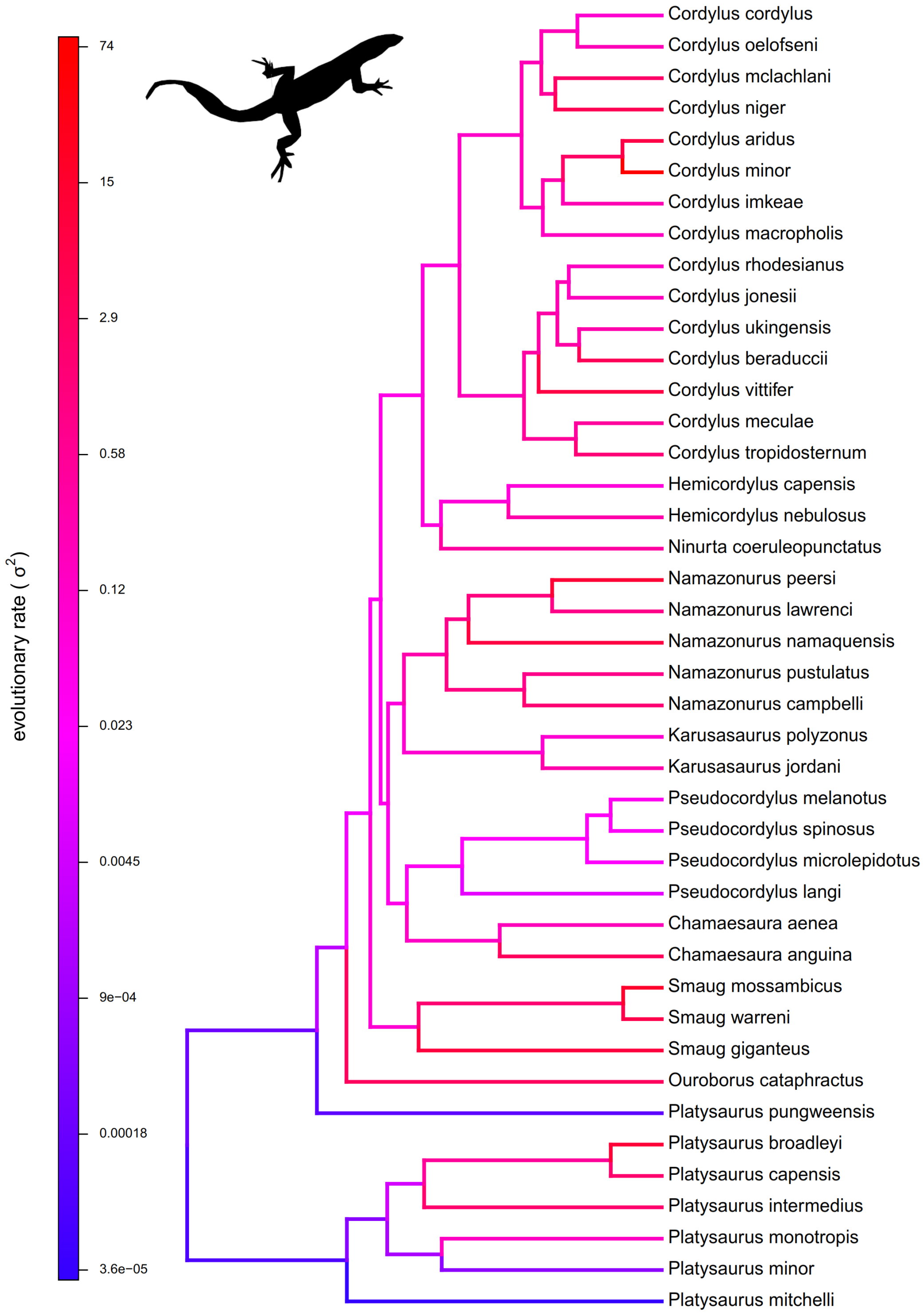

Brightness evolution rates in Cordylidae. The evolutionary rates are plotted along the edges of the phylogeny under a process of geometric Brownian evolution. Warmer colors indicate faster evolutionary rates, whereas cooler colors slower rates. Silhouette from phylopic.org.

Supplementary Figure 20

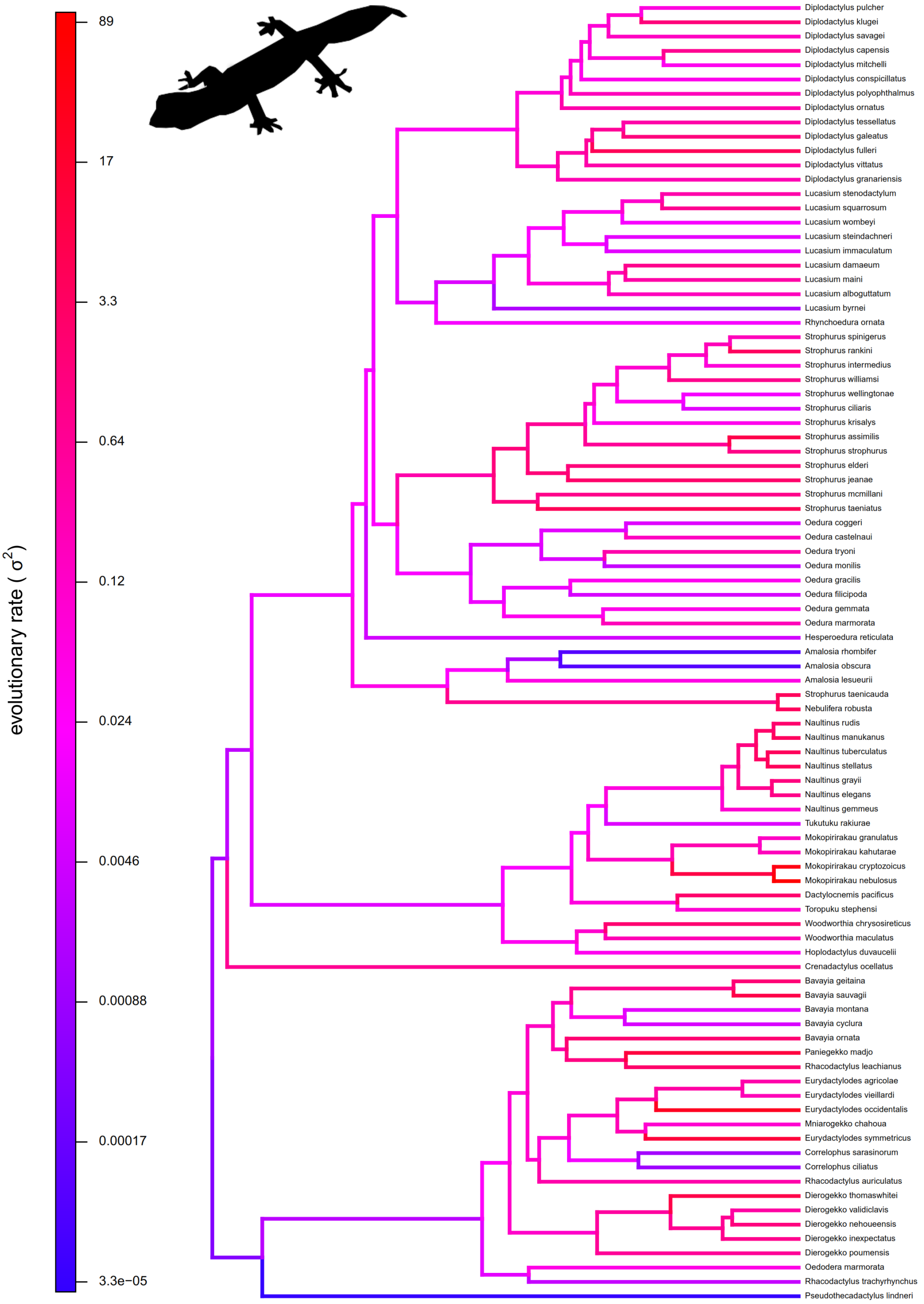

Brightness evolution rates in Diplodactylidae The evolutionary rates are plotted along the edges of the phylogeny under a process of geometric Brownian evolution. Warmer colors indicate faster evolutionary rates, whereas cooler colors slower rates. Silhouette from phylopic.org.

Supplementary Figure 21

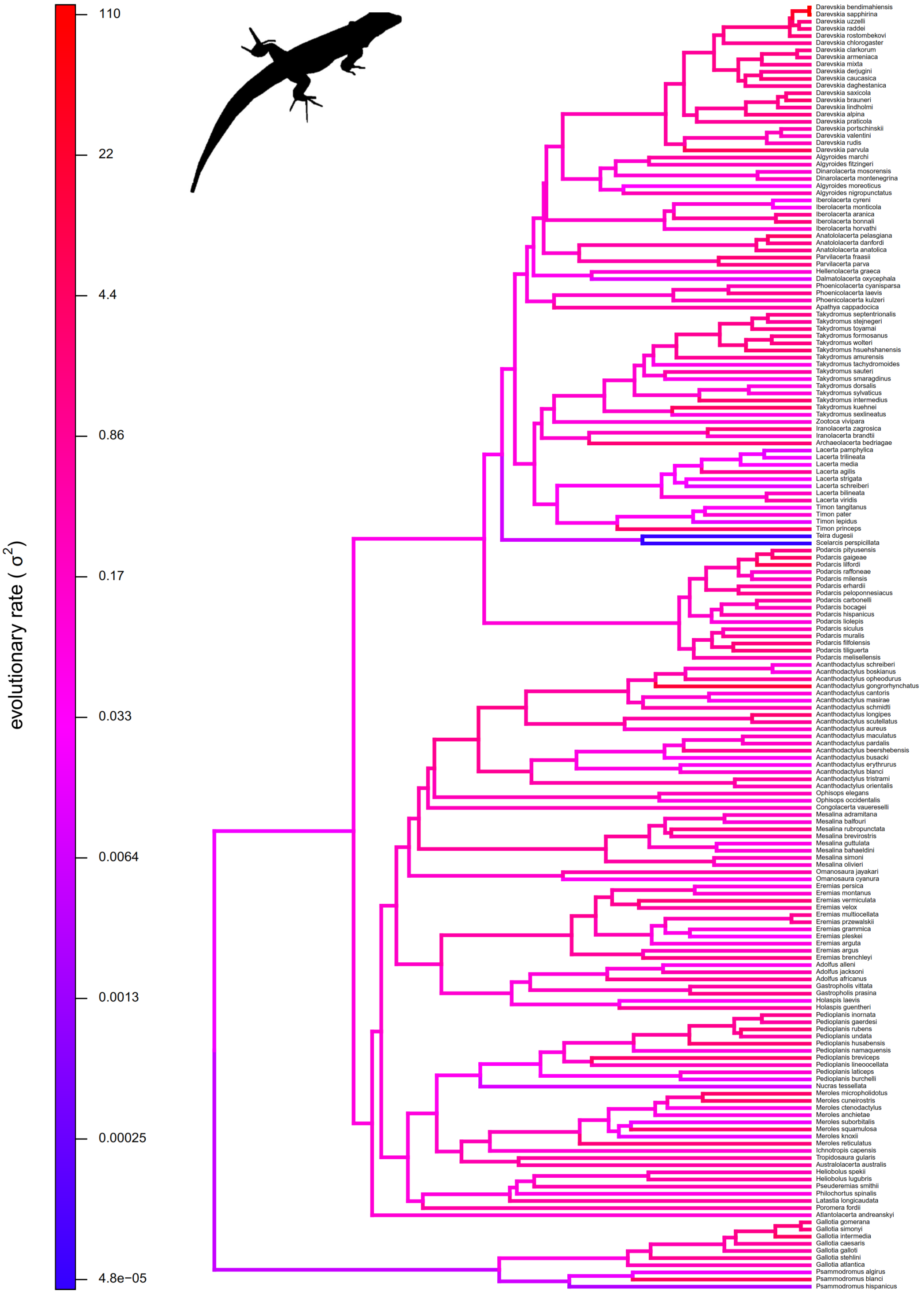

Brightness evolution rates in Lacertidae. The evolutionary rates are plotted along the edges of the phylogeny under a process of geometric Brownian evolution. Warmer colors indicate faster evolutionary rates, whereas cooler colors slower rates. Silhouette from phylopic.org.

Supplementary Figure 22

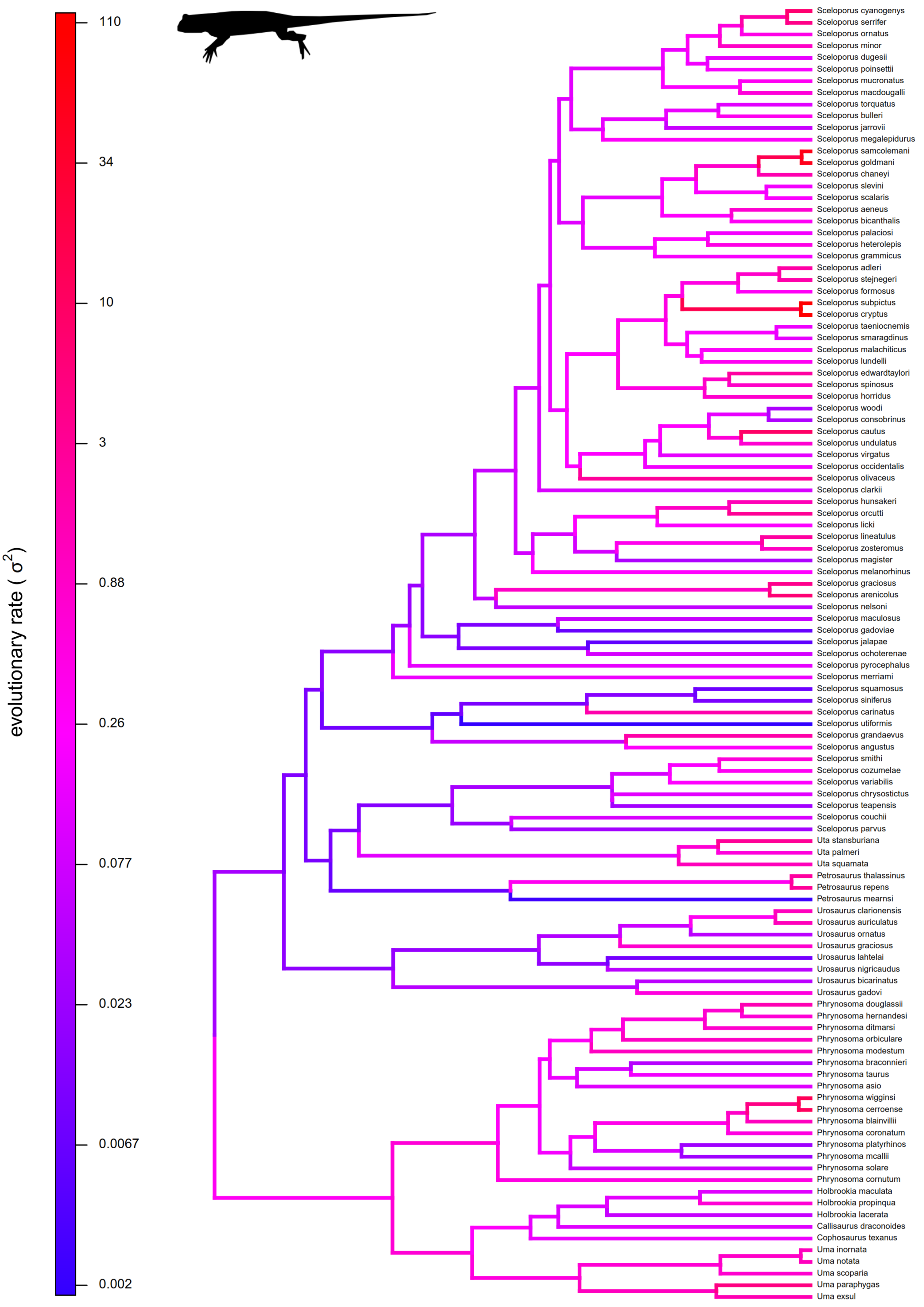

Brightness evolution rates in Phrynosomatidae. The evolutionary rates are plotted along the edges of the phylogeny under a process of geometric Brownian evolution. Warmer colors indicate faster evolutionary rates, whereas cooler colors slower rates. Silhouette from phylopic.org.

Supplementary Figure 23

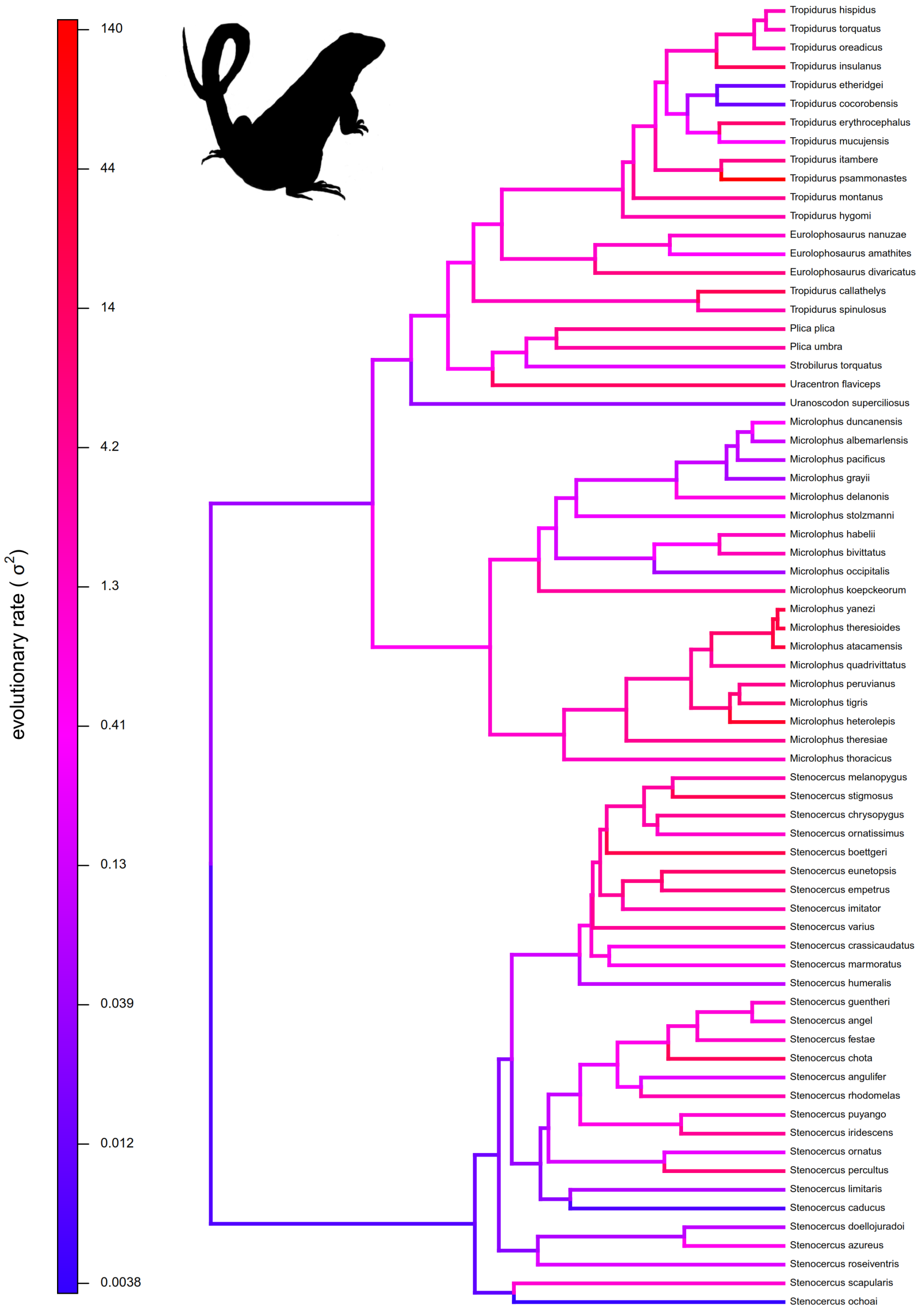

Brightness evolution rates in Tropiduridae. The evolutionary rates are plotted along the edges of the phylogeny under a process of geometric Brownian evolution. Warmer colors indicate faster evolutionary rates, whereas cooler colors slower rates. Silhouette from phylopic.org.

Supplementary Figure 24

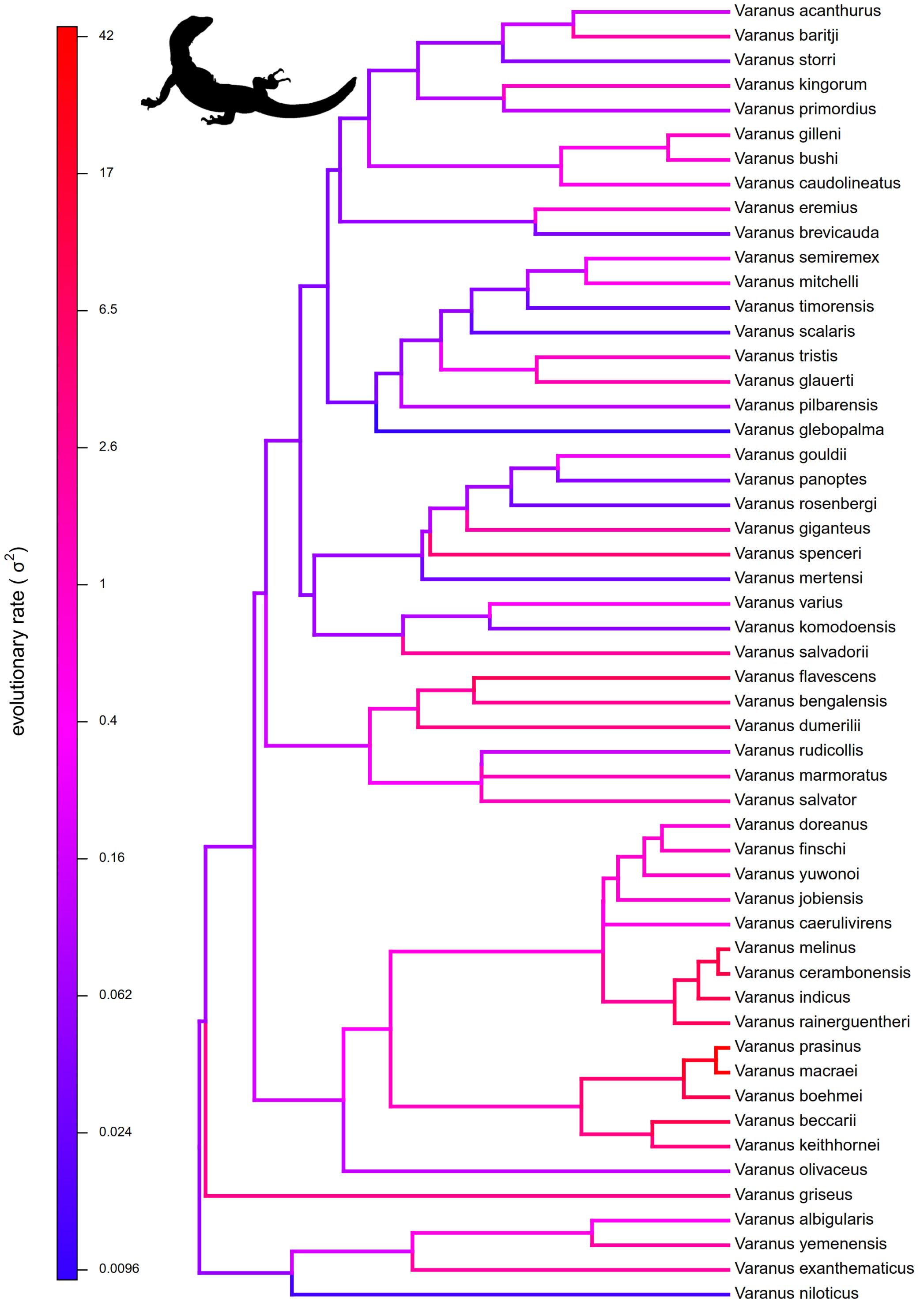

Brightness evolution rates in Varanidae. The evolutionary rates are plotted along the edges of the phylogeny under a process of geometric Brownian evolution. Warmer colors indicate faster evolutionary rates, whereas cooler colors slower rates. Silhouette from phylopic.org.

Supplementary Figure 25

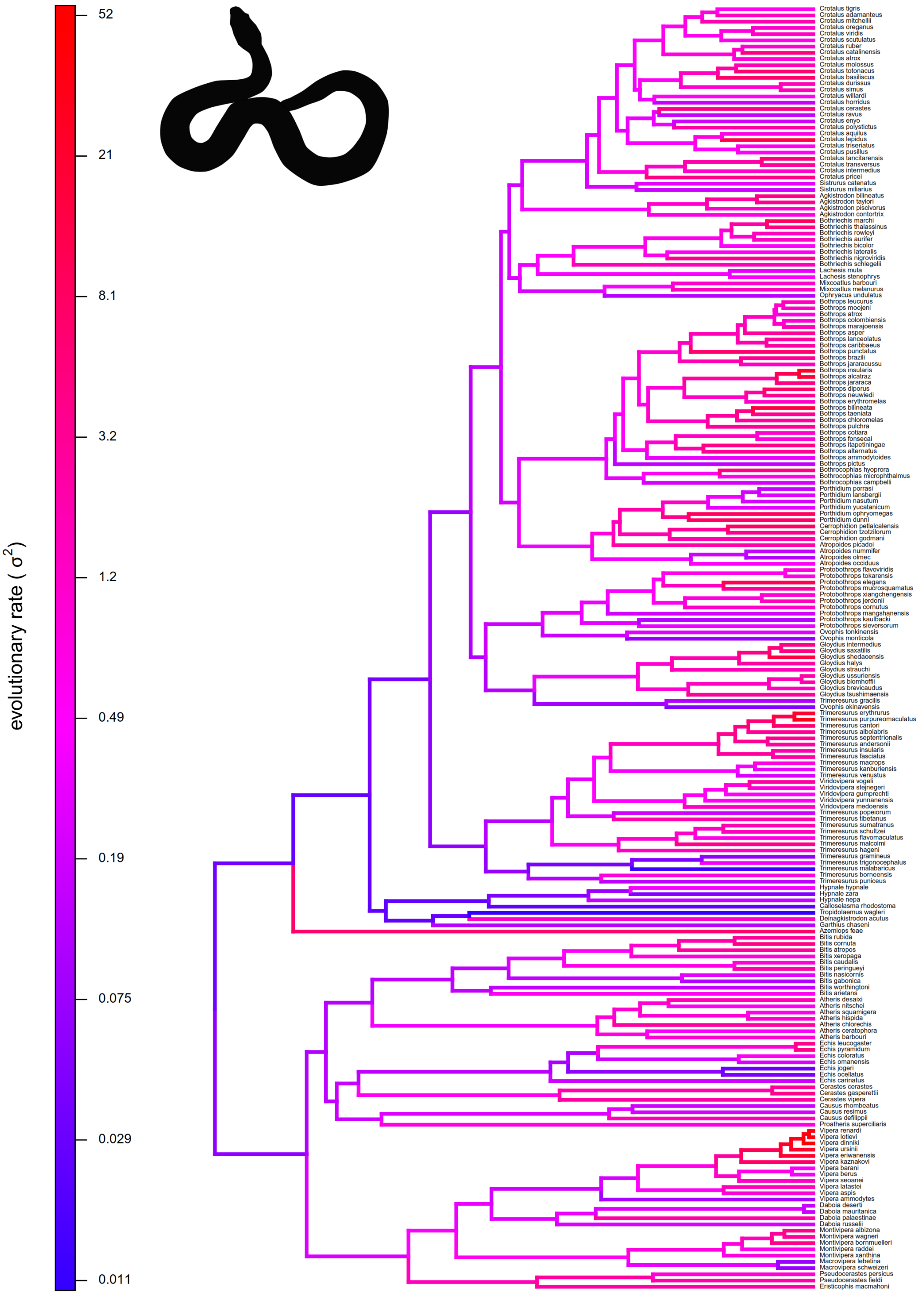

Brightness evolution rates in Viperidae. The evolutionary rates are plotted along the edges of the phylogeny under a process of geometric Brownian evolution. Warmer colors indicate faster evolutionary rates, whereas cooler colors slower rates. Silhouette from phylopic.org.

Supplementary Figure 26

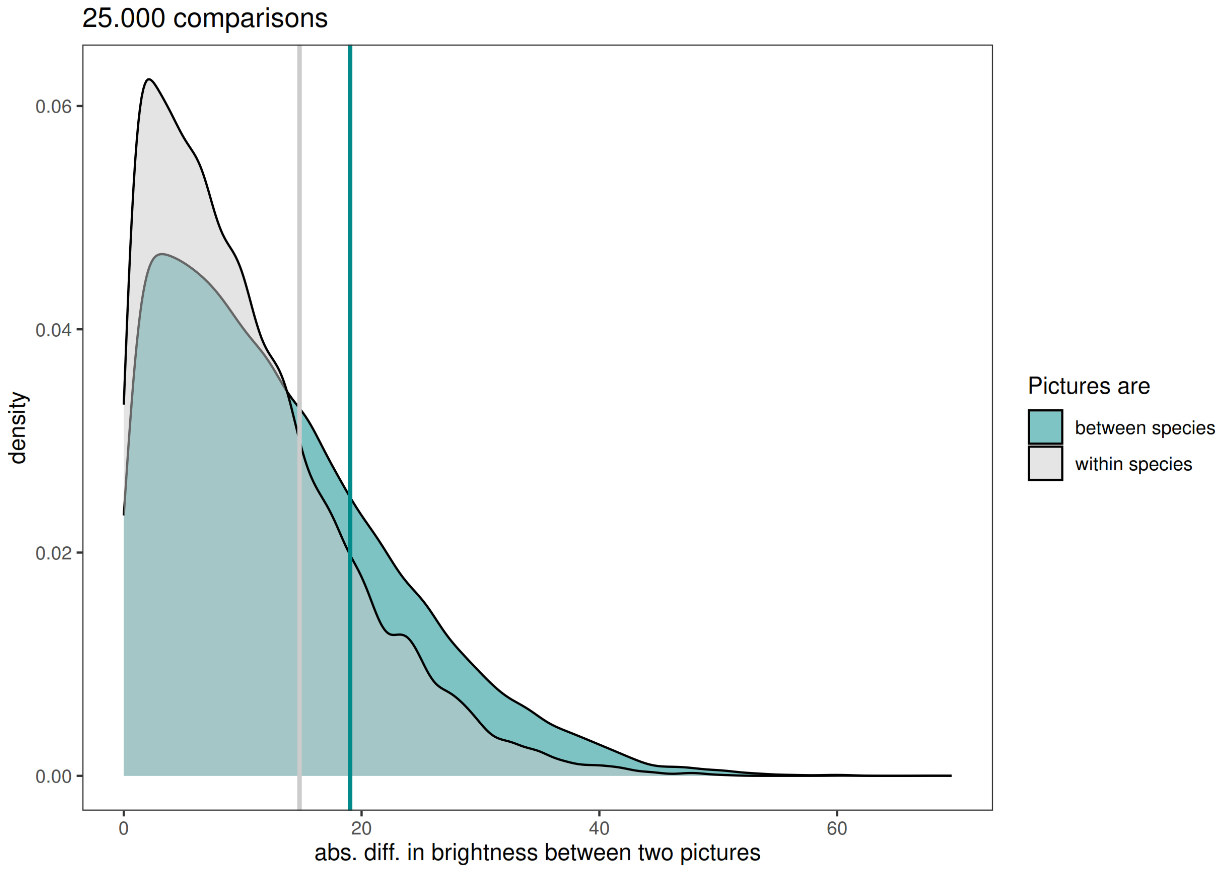

Dorsal brightness comparisons within and among species. To estimate how brightness varies among and within species, we performed 25000 comparisons contrasting two images that were either from the same or different species. Vertical lines show 75% quantiles for each group. Density indicates the percentage of absolute difference (abs. diff.) in brightness between two pictures.

Supplementary Figure 27

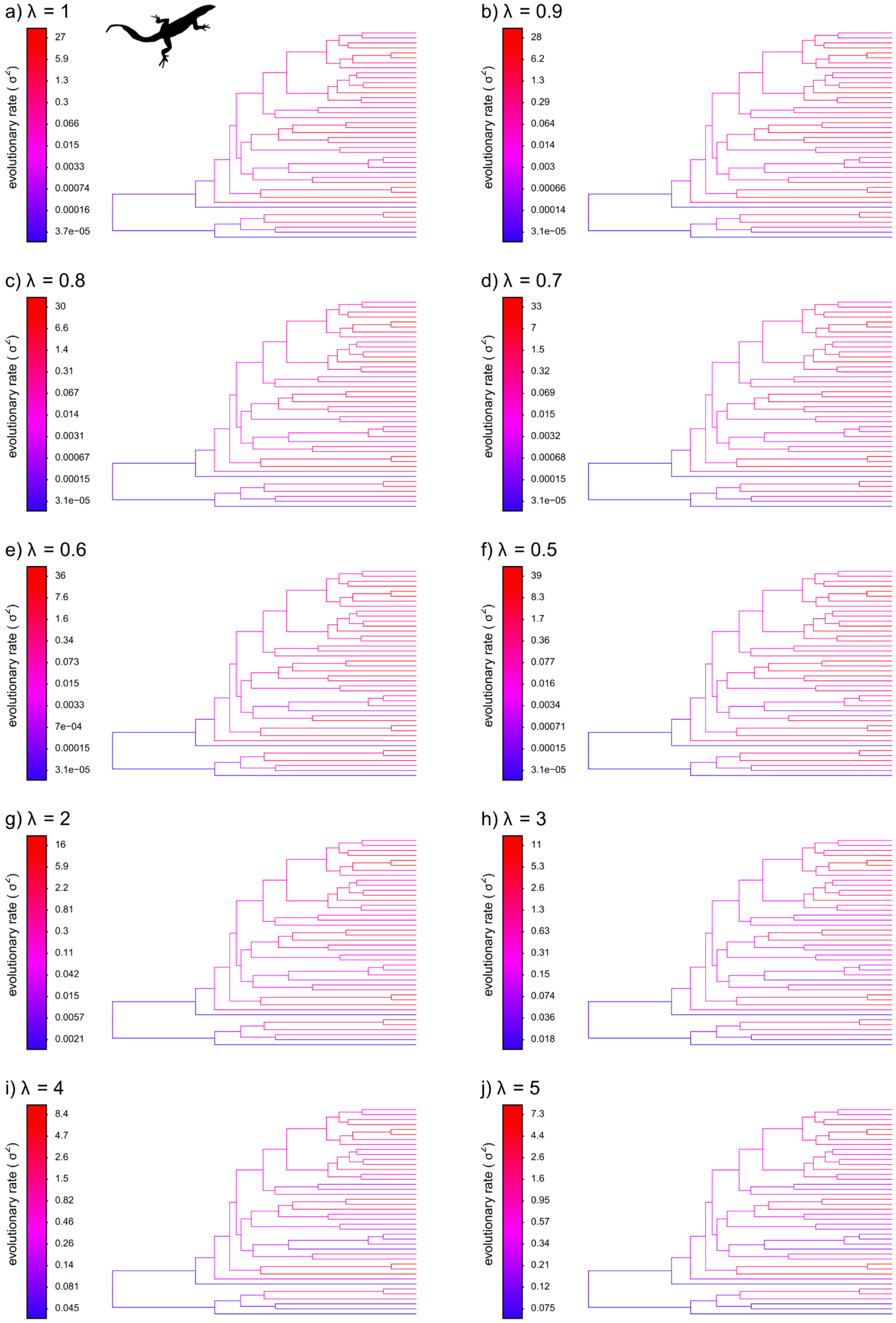

Effect of smoothing parameters in Cordylidae. The evolutionary rates of brightness are plotted under multiple smoothing parameters (λ). Results are consistent under different λ values. Silhouette from phylopic.org.

Supplementary Figure 28

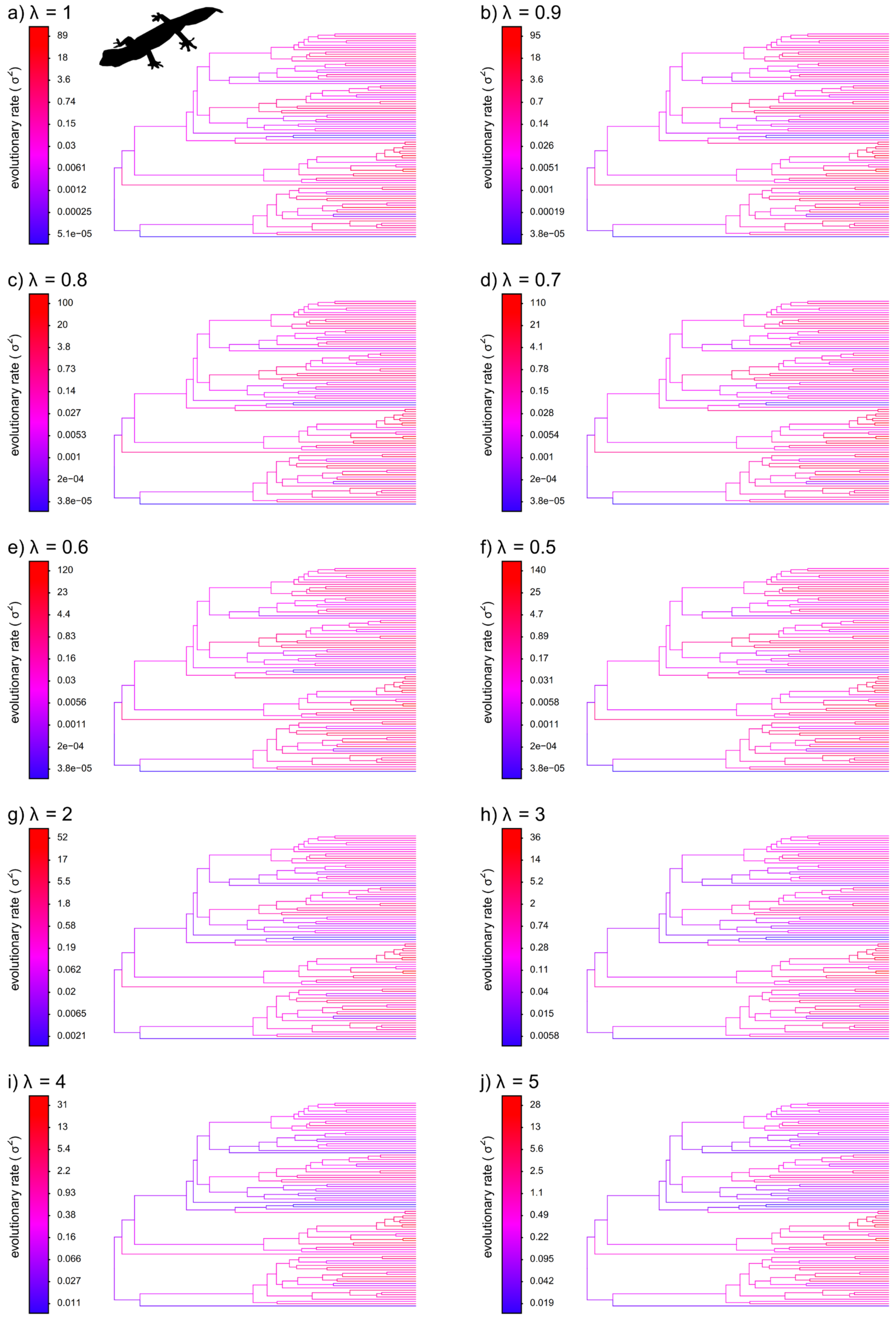

Effect of smoothing parameters in Diplodactylidae. The evolutionary rates of brightness are plotted under multiple smoothing parameters (λ). Results are consistent under different λ values. Silhouette from phylopic.org.

Supplementary Figure 29

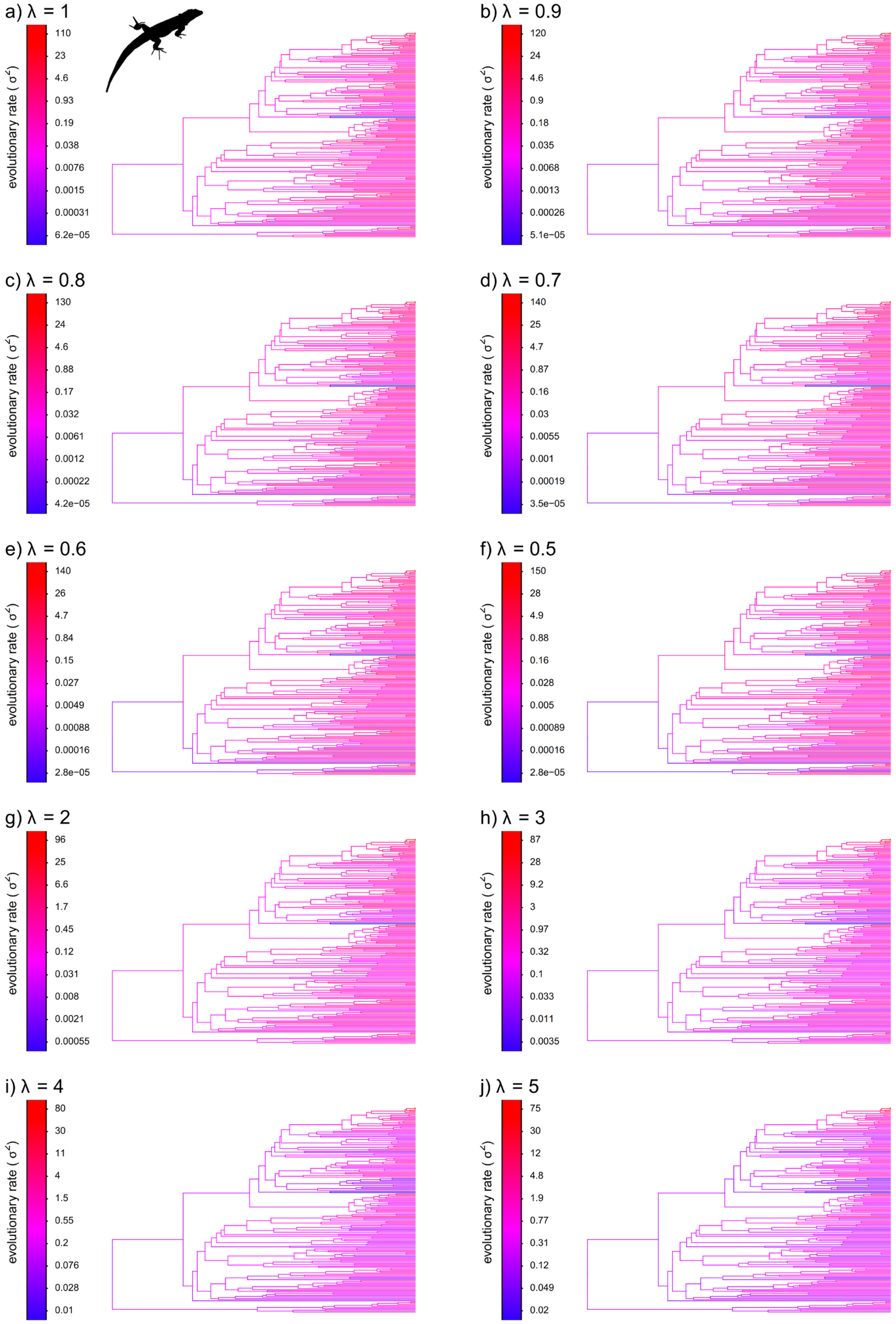

Effect of smoothing parameters in Lacertidae. The evolutionary rates of brightness are plotted under multiple smoothing parameters (λ). Results are consistent under different λ values. Silhouette from phylopic.org.

Supplementary Figure 30

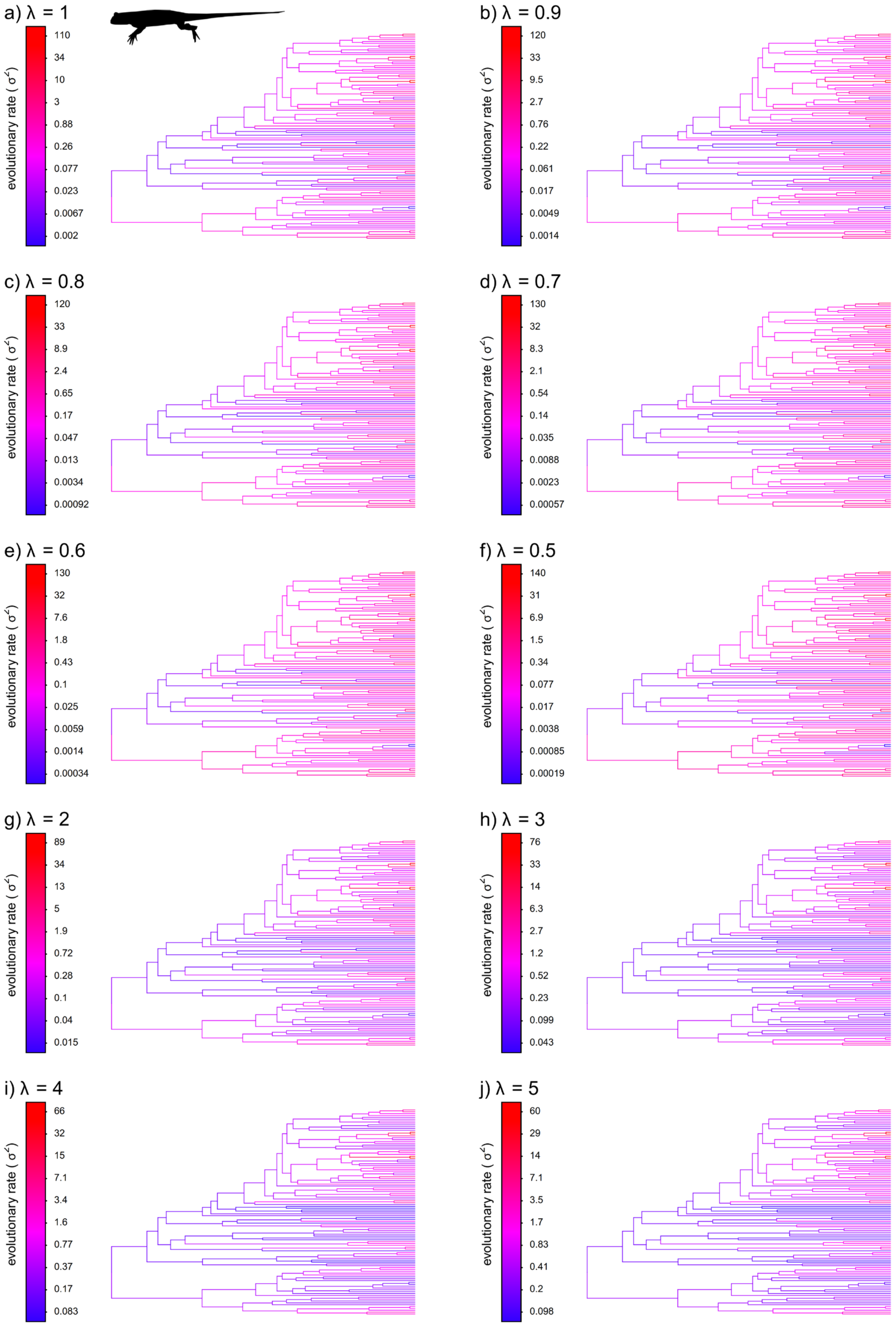

Effect of smoothing parameters in Phrynosomatidae. The evolutionary rates of brightness are plotted under multiple smoothing parameters (λ). Results are consistent under different λ values. Silhouette from phylopic.org.

Supplementary Figure 31

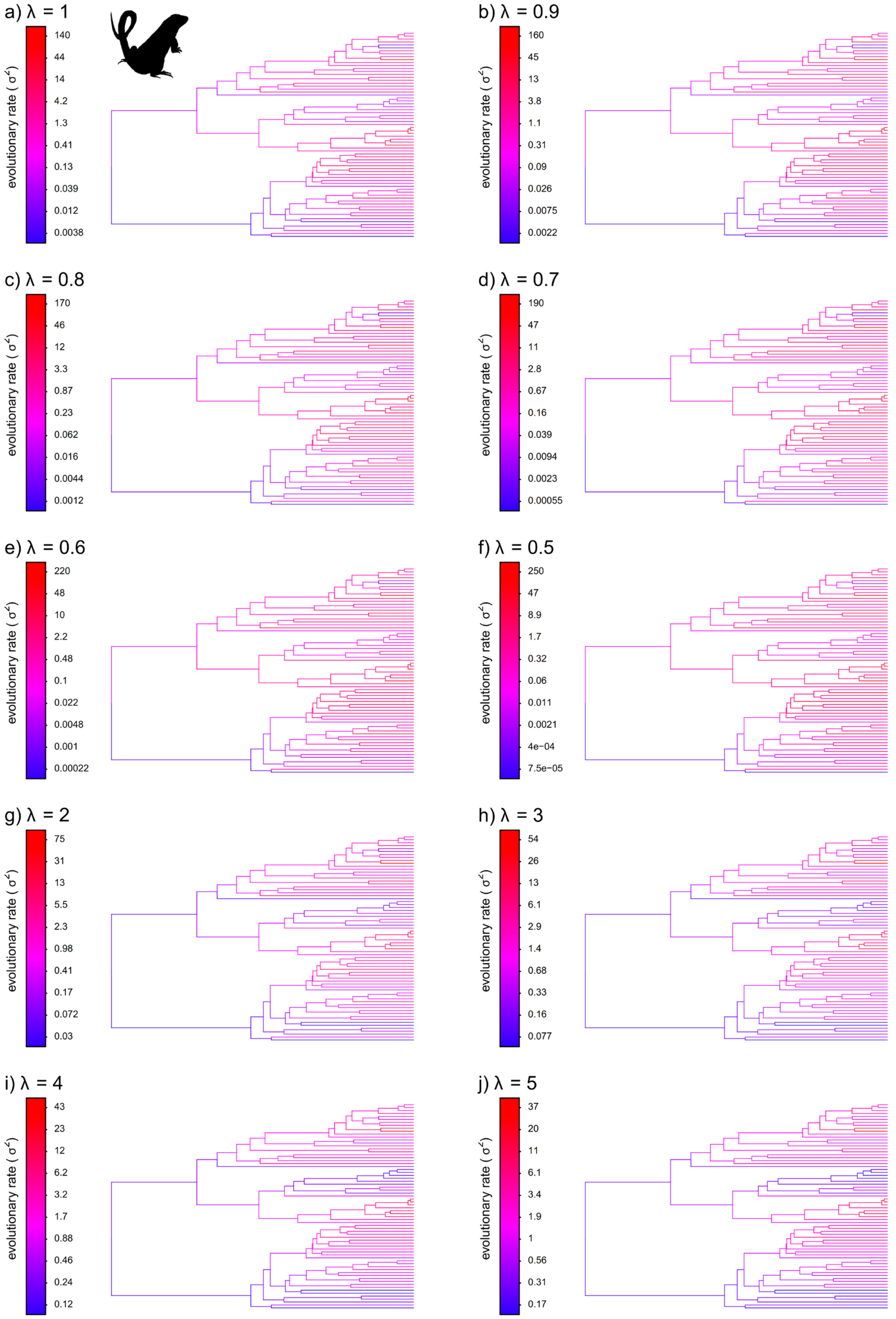

Effect of smoothing parameters in Tropiduridae. The evolutionary rates of brightness are plotted under multiple smoothing parameters (λ). Results are consistent under different λ values. Silhouette from phylopic.org.

Supplementary Figure 32

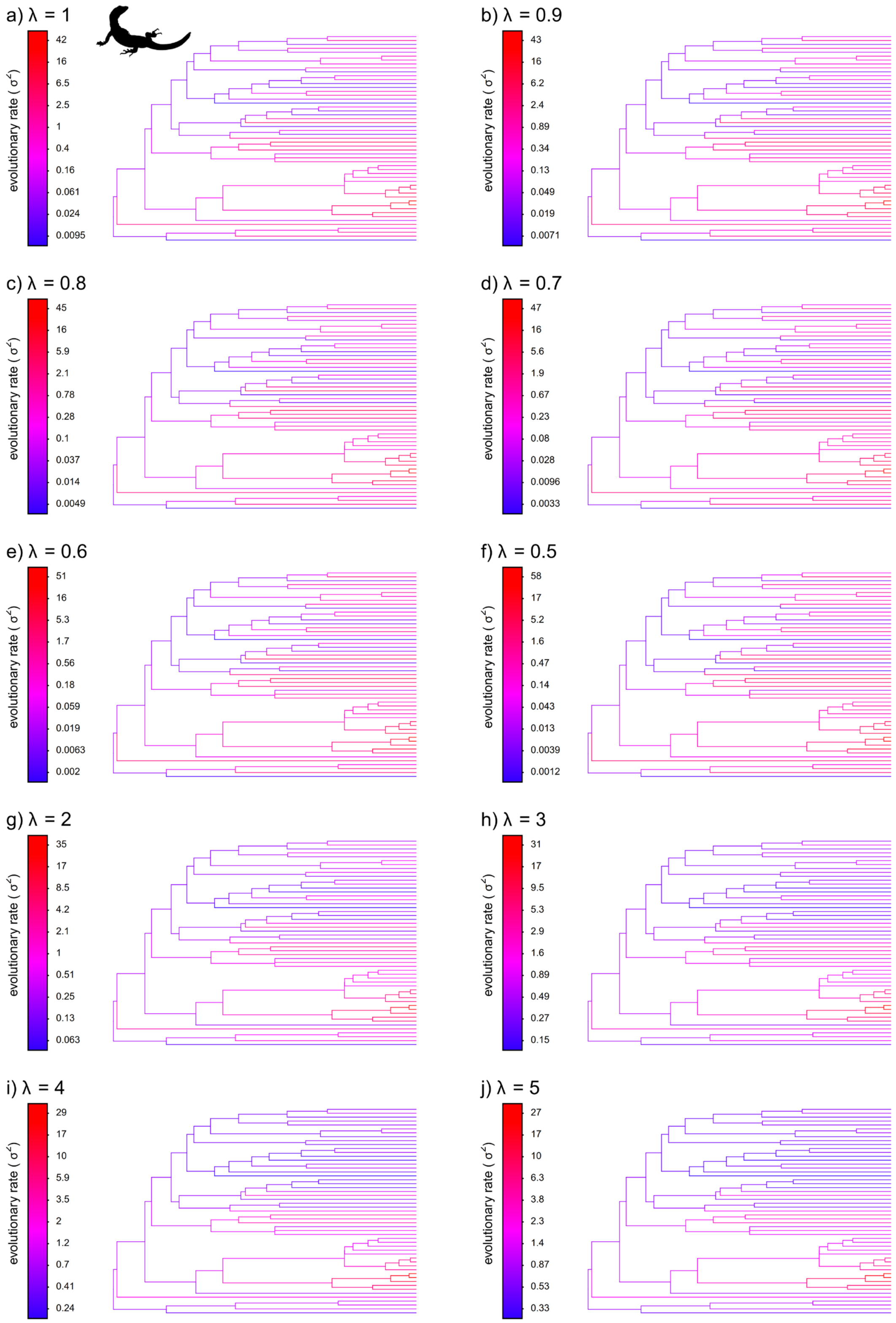

Effect of smoothing parameters in Varanidae. The evolutionary rates of brightness are plotted under multiple smoothing parameters (λ). Results are consistent under different λ values. Silhouette from phylopic.org.

Supplementary Figure 33

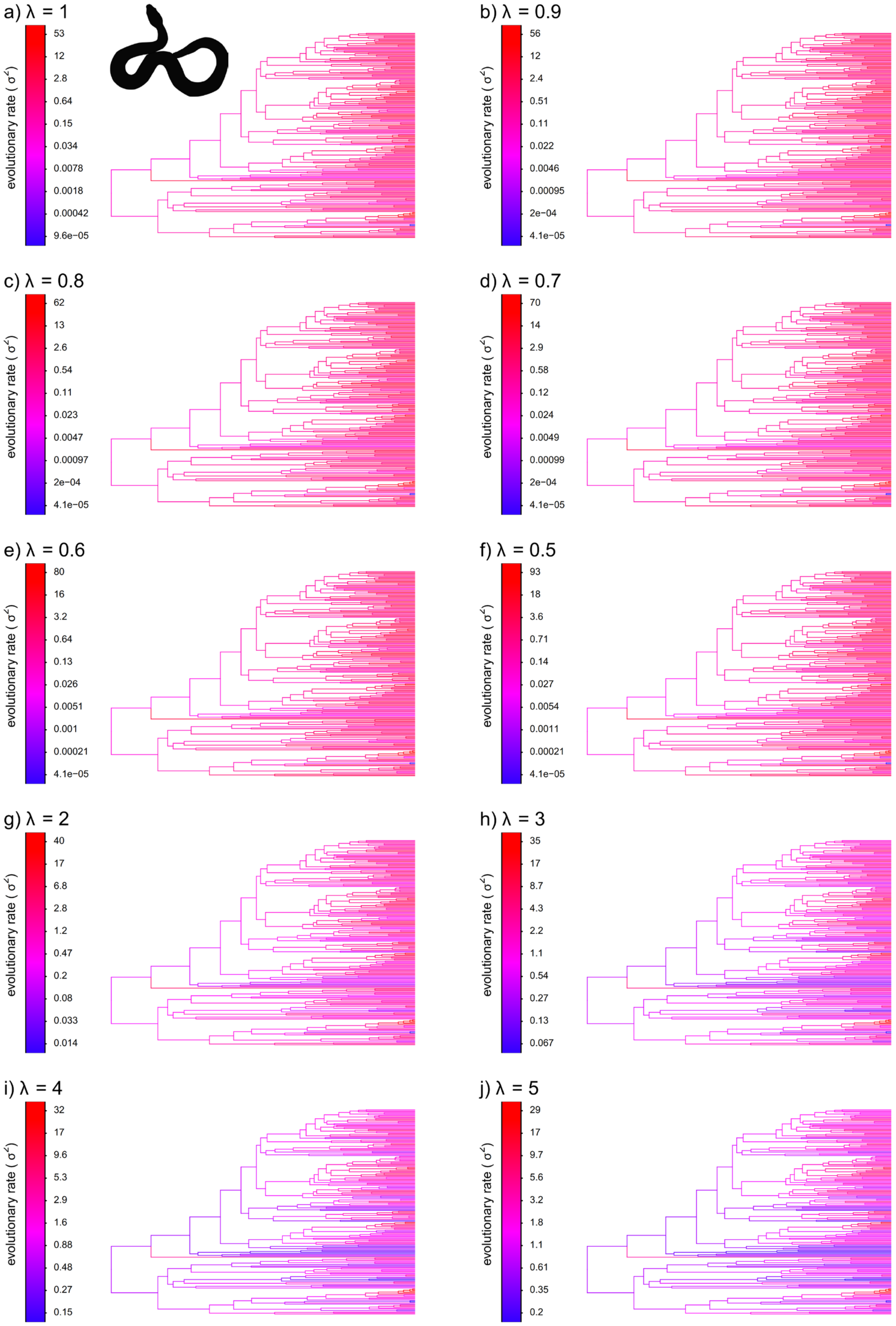

Effect of smoothing parameters in Viperidae. The evolutionary rates of brightness are plotted under multiple smoothing parameters (λ). Results are consistent under different λ values. Silhouette from phylopic.org.

Supplementary Figure 34

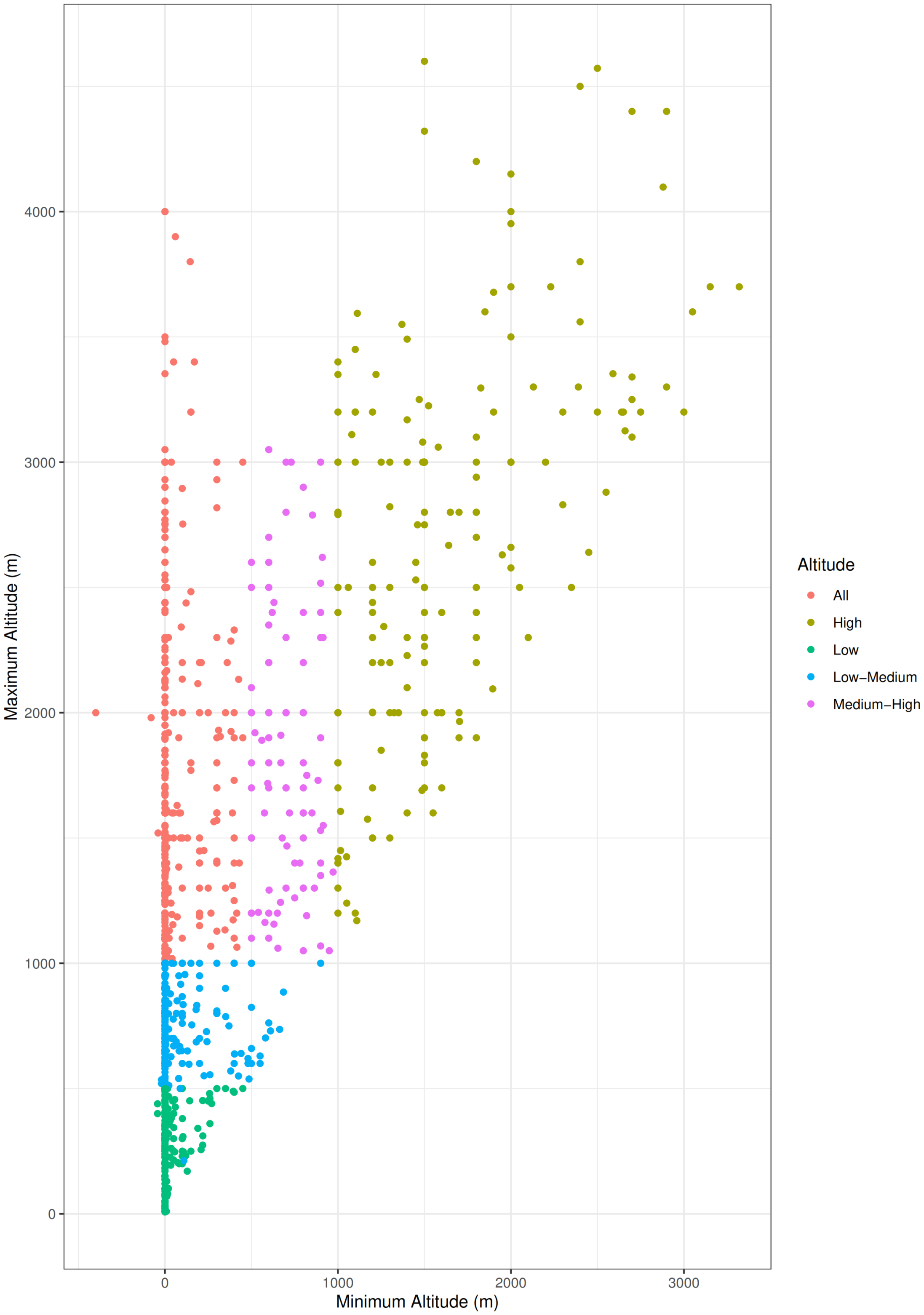

Altitude clustering. We classified altitudinal ranges as "Low" ($x \leq 500$ m), "Low-Medium" ($x \leq 1000$ m), "Medium-High" ($x > 500$ m $\land$ $x > 1000$ m), "High" ($x > 1000$ m), "All" (all the range) following established threshold classes[1].

Supplementary Figure 35

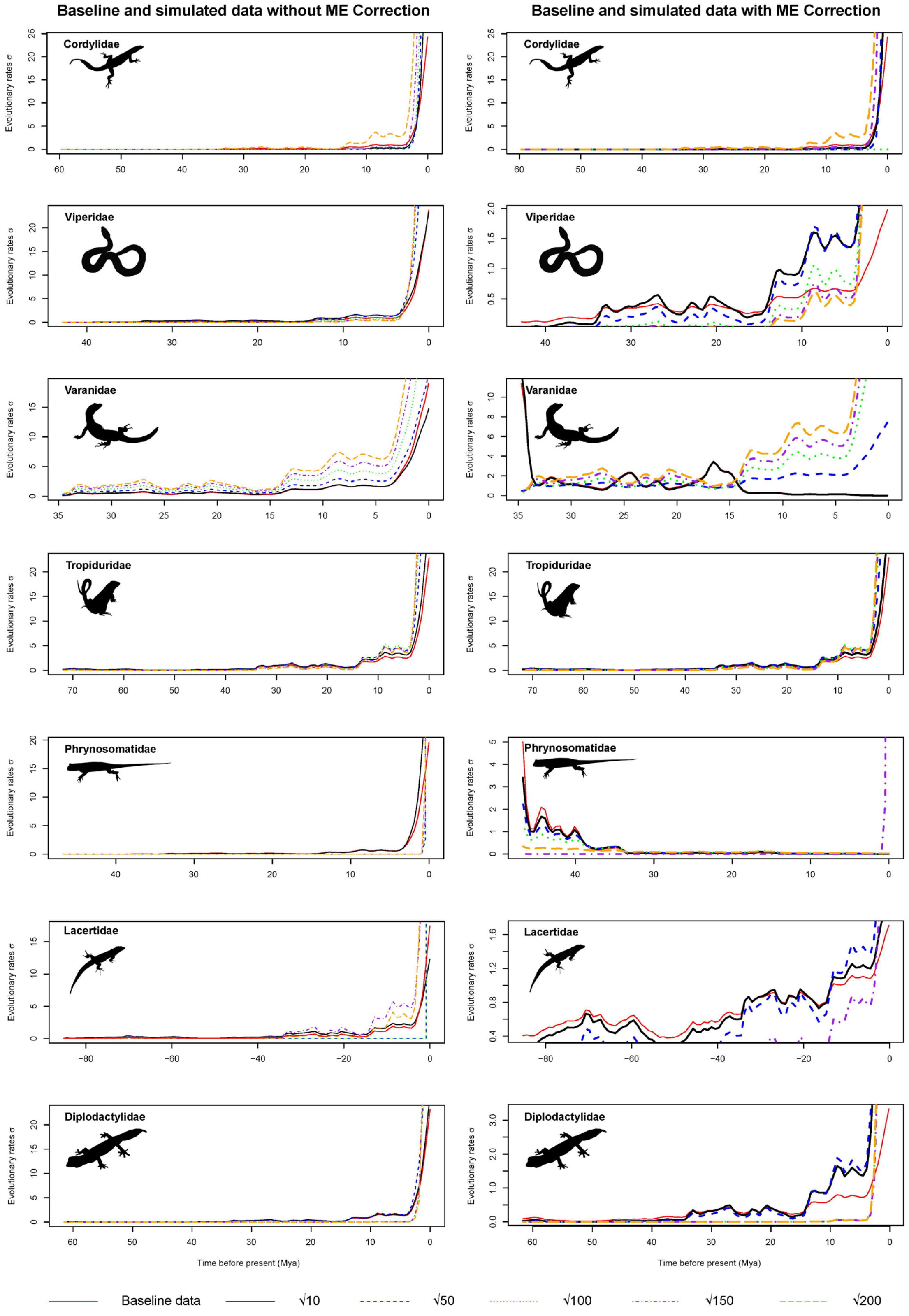

Evolutionary rate analyses. Here we fit a model of trait evolution for which evolutionary rates depend on $\delta^{18}O$ fluctuations under different simulated noises (from √10 to √200) and measurement errors. Results are consistent with other analyses (Fig. 5 and Supplementary Figure 16-18) showing that the increase in $\delta^{18}O$, and subsequent aridification period, is linked with an increase in brightness evolutionary rates. Silhouette from phylopic.org.

Supplementary Table 1

Evolution rate comparisons. Post-hoc test of brightness evolution rate estimates between trees of the seven focal groups using a two-sided, flexible Brownian multi-rate model with penalized likelihood, adjusting for multiple tests following the holm method.

			t	df	*P*	
Varanidae	vs.	Viperidae	-0.1471	58.374	1	
Varanidae	vs.	Lacertidae	0.191	58.97	1	
Varanidae	vs.	Cordylidae	0.5189	382.4961	1	
Varanidae	vs.	Diplodactylidae	2.5828	77.8755	0.1935	
Varanidae	vs.	Phrynosomatidae	4.2316	59.7604	0.0016	**
Varanidae	vs.	Tropiduridae	3.7377	61.3657	0.0078	**
Viperidae	vs.	Lacertidae	1.1719	70.2422	1	
Viperidae	vs.	Cordylidae	1.9644	159.6461	0.6521	
Viperidae	vs.	Diplodactylidae	1.4816	163.7865	1	
Viperidae	vs.	Phrynosomatidae	-2.0487	88.1176	0.6521	
Viperidae	vs.	Tropiduridae	-1.4373	96.8027	1	
Lacertidae	vs.	Cordylidae	-1.6712	144.8922	1	
Lacertidae	vs.	Diplodactylidae	-2.0281	147.512	0.6521	
Lacertidae	vs.	Phrynosomatidae	-4.5187	134.6466	0.0003	***
Lacertidae	vs.	Tropiduridae	-3.0009	180.9612	0.0553	.
Cordylidae	vs.	Diplodactylidae	0.382	94.537	1	
Cordylidae	vs.	Phrynosomatidae	0.6896	89.2701	1	
Cordylidae	vs.	Tropiduridae	0.2886	90.94	1	
Diplodactylidae	vs.	Phrynosomatidae	-2.5749	107.688	0.1935	
Diplodactylidae	vs.	Tropiduridae	-0.8867	118.1046	1	
Phrynosomatidae	vs.	Tropiduridae	1.9968	164.6472	0.6521	

Signif. codes: 0 ‘***’ 0.001 ‘**’ 0.01 ‘*’ 0.05 ‘.’ 0.1 ‘ ’ 1

Supplementary Table 2

Model evaluation. Here we compare model subsets to show the robustness of our results (Tab. 1 in main text). Two asterisks (**) indicate strongly supported parameters (variable present in all mostly supported models (DIC < 5) and has a cumulative Akaike weight of >0.75); One asterisk (*) less strongly supported (variable present in any of the mostly supported models (DIC < 5) and has a cumulative Akaike weight of > 0.75). "+" indicates a positive relationship between the variable of interest and brightness. "−" shows a negative relationship between the variable of interest and brightness. "~" displays no clear trend between the variable of interest and brightness. Altitude (**Alt**): category from low-to-high altitudes; Circadian rhythm (**Circ**): category from day-to-night activity patterns; Habitat openness (**Hab**): category from closed-to-open habitats; Body mass (**Mass**): continuous from small-to-large species; Latitude (**Lat**): category from low-to-high latitudes; Concealment (**Conc**): category conspicuous or cryptic. In all analyses we included polymorphism (binary state: Yes, No) as random effect to account for intraspecific color variation. For visualization purposes we omitted non-significant interactions such as between habitat openness and body mass. Numerical values in parenthesis show number of species analyzed within each model. Empty cells denote a lack of significant correlation. Double dots '**:**' indicates an interaction. Alencar *et al.*: viper phylogeny from Alencar *et al.*[2]; Stanley *et al.*: cordylid phylogeny from Stanley *et al.*[3]

**Model**	**Alt**	**Circ**	**Hab**	**Mass**	**Lat**	**Alt:Hab**	**Conc:Hab**	**Lat:Mass**	**Alt:Mass**
Total<br>(1249)	~**	-**	+**	-**	~**	-**	~**		
Total<br>≥5 images<br>(1033)	~*	-**	+**	-*	~*		~**		
Total<br>w/o fossorial<br>(1164)	~**	-**	+**	-*	~**	-*	~**		
Total<br>OU model<br>(1249)	~**	-**	+**	-*	~**	-**	~**		
Bootstrap 1<br>(1033)		-**	+**	-*	~*		~**		
Bootstrap 2<br>(1033)		-**	+**	-*	~**		~**		
Bootstrap 3<br>(1033)		-**	+**	-**	~*		~**		
Viperidae<br>(206)			+**						
Viperidae<br>Alencar *et al.*<br>(261)			+*						
Cordylidae<br>(42)	~**		+*	-**					~**
Cordylidae<br>Stanley *et al.*<br>(49)	~*		+*	-*	-**			~*	

Supplementary References

1. Gaskell, D. E. *et al.* The latitudinal temperature gradient and its climate dependence as inferred from foraminiferal δ18O over the past 95 million years. *Proc. Natl. Acad. Sci. U. S. A.* **119**, e2111332119 (2022).
2. Alencar, L. R. V. *et al.* Diversification in vipers: Phylogenetic relationships, time of divergence and shifts in speciation rates. *Mol. Phylogenet. Evol.* **105**, 50–62 (2016).
3. Stanley, E. L., Bauer, A. M., Jackman, T. R., Branch, W. R. & Mouton, P. L. F. N. Between a rock and a hard polytomy: Rapid radiation in the rupicolous girdled lizards (Squamata: Cordylidae). *Mol. Phylogenet. Evol.* **58**, 53–70 (2011).
