# Supplementary material for: Habitat openness and squamate color evolution over deep time
Source: Nat Commun. 2025 Mar 17;16:2625. doi: 10.1038/s41467-025-57547-6 (PMC11914068; doi:10.1038/s41467-025-57547-6)
Supplement: Supplementary file 2 — Reporting Summary [file 41467_2025_57547_MOESM2_ESM.pdf]

nature portfolio

Corresponding author(s): Jonathan Goldenberg
jonathan.goldenberg@ugent.be

Last updated by author(s): Feb 20, 2025

Reporting Summary

Nature Portfolio wishes to improve the reproducibility of the work that we publish. This form provides structure for consistency and transparency in reporting. For further information on Nature Portfolio policies, see our Editorial Policies and the Editorial Policy Checklist.

Statistics

For all statistical analyses, confirm that the following items are present in the figure legend, table legend, main text, or Methods section.

n/a	Confirmed	
☐	☒	The exact sample size (*n*) for each experimental group/condition, given as a discrete number and unit of measurement
☐	☒	A statement on whether measurements were taken from distinct samples or whether the same sample was measured repeatedly
☐	☒	The statistical test(s) used AND whether they are one- or two-sided<br>*Only common tests should be described solely by name; describe more complex techniques in the Methods section.*
☐	☒	A description of all covariates tested
☐	☒	A description of any assumptions or corrections, such as tests of normality and adjustment for multiple comparisons
☐	☒	A full description of the statistical parameters including central tendency (e.g. means) or other basic estimates (e.g. regression coefficient) AND variation (e.g. standard deviation) or associated estimates of uncertainty (e.g. confidence intervals)
☐	☒	For null hypothesis testing, the test statistic (e.g. *F*, *t*, *r*) with confidence intervals, effect sizes, degrees of freedom and *P* value noted<br>*Give P values as exact values whenever suitable.*
☐	☒	For Bayesian analysis, information on the choice of priors and Markov chain Monte Carlo settings
☒	☐	For hierarchical and complex designs, identification of the appropriate level for tests and full reporting of outcomes
☐	☒	Estimates of effect sizes (e.g. Cohen's *d*, Pearson's *r*), indicating how they were calculated

Our web collection on statistics for biologists contains articles on many of the points above.

Software and code

Policy information about availability of computer code

Data collection	Brightness data have been measured with MacroBright v.0.1 (Goldenberg et al. 2021, DOI: 10.1038/s42003-020-01524-w) in ImageJ 1.53q using online photographic data. All references are provided in our dataset. All other analyzed variables have been collected from the literature for which all references are indicated in our dataset. The dataset is deposited at our provided repository.
Data analysis	All statistical analyses have been perfomerd in R v.4.1.3. All R-scripts and data are deposited at our provided repository.

For manuscripts utilizing custom algorithms or software that are central to the research but not yet described in published literature, software must be made available to editors and reviewers. We strongly encourage code deposition in a community repository (e.g. GitHub). See the Nature Portfolio guidelines for submitting code & software for further information.

Data

Policy information about availability of data

All manuscripts must include a data availability statement. This statement should provide the following information, where applicable:

- Accession codes, unique identifiers, or web links for publicly available datasets
- A description of any restrictions on data availability
- For clinical datasets or third party data, please ensure that the statement adheres to our policy

All data associated with this manuscript, including all its source data, are available on Zenodo: https://doi.org/10.5281/zenodo.14866945

Research involving human participants, their data, or biological material

Policy information about studies with human participants or human data. See also policy information about sex, gender (identity/presentation), and sexual orientation and race, ethnicity and racism.

Reporting on sex and gender	No gender or sex data has been collected in this study, as our assessment focused on macroscale variations across species
Reporting on race, ethnicity, or other socially relevant groupings	No race, ethnicity, or other socially relevant groupings have been considered in this study
Population characteristics	There are no population characteristics to be considered in this study
Recruitment	No participant has been recruited in this study
Ethics oversight	All data were obtained from the literature; therefore, no ethical approval was required

Note that full information on the approval of the study protocol must also be provided in the manuscript.

Field-specific reporting

Please select the one below that is the best fit for your research. If you are not sure, read the appropriate sections before making your selection.

☐ Life sciences ☐ Behavioural & social sciences ☒ Ecological, evolutionary & environmental sciences

For a reference copy of the document with all sections, see nature.com/documents/nr-reporting-summary-flat.pdf

Ecological, evolutionary & environmental sciences study design

All studies must disclose on these points even when the disclosure is negative.

Study description	Using a global dataset of 1249 squamates, we evaluate whether and how six key eco-environmental variables (vegetation structure, body mass, cirdian rhythm, latitudinal distribution, altitude variation, concelment) and their interactions shaped the evolutionary history of their coloration.
Research sample	Global dataset of 1249 squamates (Squamata Oppel, 1811)
Sampling strategy	We initially collected information on 1372 taxonomically unambiguous species (12 % of the order) from 25 families covering major groups and capturing different ecological strategies and evolutionary paths; but as images of aquatic species (n = 33) could not be consistent due to different light conditions, and we could not retrieve image data for 18, we ultimately analyzed 1321 species.
Data collection	Data have been collected by JG, KB, and students supervised by JG
Timing and spatial scale	Data collection spanned 3.5 years: July 2019 - January 2023
Data exclusions	We excluded aquatic species (n = 33) beacause light conditions are not consistent in such environments.
Reproducibility	All analyses and results are fully reproducible with our provided R script and dataset
Randomization	We produced model substets to evaluate to robustenss of our results.
Blinding	Analyses did not make use of blinding because data collection used either analytical tools or literature-based information

Did the study involve field work? ☐ Yes ☒ No

Reporting for specific materials, systems and methods

We require information from authors about some types of materials, experimental systems and methods used in many studies. Here, indicate whether each material, system or method listed is relevant to your study. If you are not sure if a list item applies to your research, read the appropriate section before selecting a response.

Materials & experimental systems

n/a	Involved in the study
☒	☐ Antibodies
☒	☐ Eukaryotic cell lines
☒	☐ Palaeontology and archaeology
☒	☐ Animals and other organisms
☒	☐ Clinical data
☒	☐ Dual use research of concern
☒	☐ Plants

Methods

n/a	Involved in the study
☒	☐ ChIP-seq
☒	☐ Flow cytometry
☒	☐ MRI-based neuroimaging
